# Seed quality as affected by intercropping of Chickpea and *L. iberica*

**DOI:** 10.1371/journal.pone.0332264

**Published:** 2025-10-30

**Authors:** Maryam Mirdoraghi, Saeideh Maleki Farahani, Alireza Rezazadeh

**Affiliations:** 1 Department of Crop Production and Plant Breeding, Faculty of Agriculture, Shahed University, Tehran, Iran; 2 Department of Plant Protection, Faculty of Agriculture, Shahed University, Tehran, Iran; Huazhong University of Science and Technology, CHINA

## Abstract

Producing high-quality seeds is a significant priority for the agricultural industry and plays a vital role in enhancing and stabilizing crop yields. However, the conditions experienced by a mother plant during seed development and maturation can significantly influence seed quality. Intercropping oilseeds with other crops, especially legumes, may contribute to a sustainable food supply and increase agricultural sustainability and resilience. Therefore, the objective of this research was to investigate the effect of intercropping on the quality of seeds from dragon’s head (*Lallemantia iberica*) and chickpea (*Cicer arietinum*) produced under different irrigation regimes and sowing dates. Seeds of chickpea and *L. iberica* were obtained from the research farm of the Agricultural College of Shahed University in Tehran, Iran, where the maternal plants were grown under: a) three irrigation regimes (irrigation after 20% (I_20_: short interval), 40% (I_40_: medium interval) of soil available water depletion, and supplementary irrigation (IS: long interval) in only two stages, including the sowing and pre-flowering stages, based on a 20% depletion of soil available water); b) the autumn sowing date on November 6 (S_1_) and the spring sowing date on March 6 (S_2_); and c) sole system and intercropping treatments included: a) sole system (Ss), and b) intercropping of 50% chickpea: 50% *L. iberica* (Ic), applied across two consecutive years, 2021–22 and 2022–23. This study found that Ic (I_20_S_1_) and Ic (I_40_S_1_) treatments improved germination indexs due to favorable maternal plant conditions. In contrast, Ic (I_S_S_2_) and Ic (I_S_S_1_) treatments increased stress markers such as hydrogen peroxide (H_2_O_2_), malondialdehyde (MDA), and electrical conductivity (EC). Intercropping also enhanced seed nutrient content ((nitrogen (N), phosphorus (P), potassium (K)) and fatty acid levels, which correlated positively with germination indexs. These findings suggest that intercropping systems, especially Ic (I_20_S_1_) and Ic (I_40_S_1_) treatments, are an effective strategy for improving seed quality, resilience to water stress, and agricultural sustainability.

## Introduction

Seed germination constitutes the critical foundation for crop establishment and yield potential [1,2]. Producing high-quality seeds—defined by genetic purity, physiological vigor, and stress resilience [3]—is paramount for global food security [4,5]. Seed quality has a significant impact on crop development and yield. High-quality seeds can substantially boost yields [6], et their quality is profoundly influenced by maternal environmental conditions. Drought and temperature extremes disrupt nutrient allocation to seeds, inducing oxidative damage that compromises membrane integrity and storage reserves [7,8]. Furthermore, seed quality is modulated by genetic factors, ecological conditions, and management practices [9], particularly intercropping systems where legumes are cultivated alongside other crops to optimize resource use during critical growth stages [10].

Intercropping, especially legume-oilseed combinations, offers strategic advantages for stress mitigation through enhanced resource partitioning (light, water, nutrients) [11], biological nitrogen fixation, and improved soil microclimate [12,13], In addition, the use of legume oilseed intercropping increases yield and reduces nitrogen fertilizer demand compared to the sole system production of legume oilseeds [11]. However, recent studies reveal significant contradictions. While cereal–legume intercropping consistently improves seed germination and weight [11,14], oilseed-based systems show inconsistent outcomes due to species-specific competition for phosphorus and water [15], often manifesting as increased lipid peroxidation that reduces unsaturated fatty acid content [16]. Although Intercropping is increasingly adopted for its ecological benefits; however, critical knowledge gaps remain regarding antioxidant defense mechanisms in companion species exposed to competitive stress [17,18].

While numerous studies have explored seed quality within intercropping systems, the majority have concentrated on cereal–legume combinations, leaving oilseed species underrepresented. For example, Rezaei-Chiyaneh et al. (2019) demonstrated enhanced essential oil content in fennel and dragon’s head under legume intercropping, yet key biochemical markers such as MDA and H_2_O_2_ were seldom evaluated [19]. Current research is constrained by methodological limitations—including an overreliance on germination indices without biochemical validation of oxidative markers such as MDA, H_2_O_2_, and fatty acids [20]; inadequate exploration of abiotic stress gradients like irrigation–sowing date interactions [21]; and a persistent research bias favoring cereals over underutilized oilseed crops like Dragon’s head (*Lallemantia iberica*), despite its proven resilience and agronomic potential [22]. Lallemantia iberica *(L. iberica)*, a valuable medicinal and oilseed crop, exhibits high adaptability to arid environments. The seeds of this species contain 30–45% oil by weight [23,24] and are rich in linolenic acid, which constitutes 67–74% of their composition. This fatty acid is associated with significant health benefits [11]. On the other hand, the demand for oilseeds and legumes has grown dramatically over the last 50 years [25], with cereals, legumes, and oilseeds covering more than 90% of global farmland [26].

This study addresses these deficiencies through comprehensive assessment of physiological, biochemical, and physical seed quality parameters in chickpea-*L. iberica* intercropping across three irrigation regimes, two sowing dates, and sole versus intercrop systems. We hypothesize that chickpea root exudates enhance seed quality and ecological stability by scavenging rhizosphere ROS [27], buffering microclimatic fluctuations [28], and stimulating rhizosphere-level P/N nutrient synergy [29]. Understanding these interactions will provide valuable insights for optimizing *L. iberica* seed production and promoting sustainable agricultural practices.

## Materials and methods

### Study site and environmental conditions

This study was conducted over two consecutive growing seasons (2021–2022 and 2022–2023) at the Research Farm of Shahed University, Tehran, Iran (35°43’N, 51°24’E; 1190 m a.s.l.), characterized by an arid to semi-arid climate. Complete meteorological data, including monthly variations in temperature, precipitation, relative humidity, and solar radiation for both growing seasons, are presented in S1 Table.

### Seed origin and mother plant production conditions of Chickpea and L. iberica

Seeds of chickpea and *L. iberica* were obtained from the research farm of the Agricultural College of Shahed University, Tehran, Iran, where the maternal plants were grown under: a) three irrigation regimes (irrigation after 20% (I_20_: short interval), 40% (I_40_: medium interval) of soil available water depletion, and supplementary irrigation (Is: long interval) in only two stages, including the sowing and pre-flowering stages, based on a 20% depletion of soil available water); b) the autumn sowing date on November 6 (S_1_) and the spring sowing date on March 6 (S_2_); and c) sole system and intercropping treatments shown in S1 Fig in S1 File: a) sole system (Ss) and b) intercropping of 50% chickpea: 50% *L. iberica* (Ic). A 1:1 planting ratio in legume–oilseed intercropping systems is frequently adopted to minimize interspecific dominance and maximize facilitative interactions. This ratio promotes symmetric resource partitioning—including light interception, water uptake, and soil nutrient absorption—thereby balancing competition and cooperation [30,31].

### Experimental design

**Fig 1:**
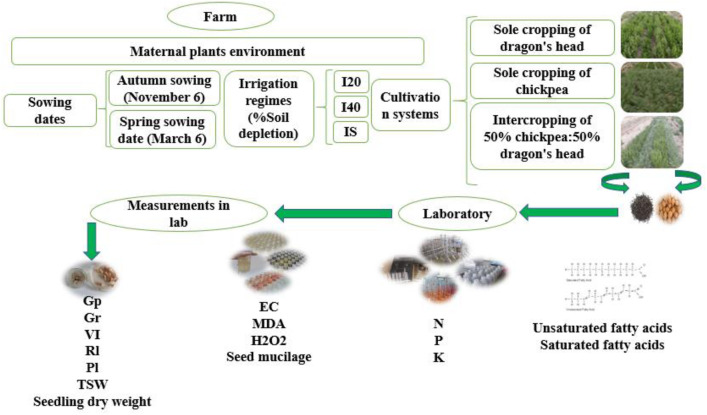
Overview of the experiment: *L. iberica* and chickpea maternal plants environment: irrigation regimes; *L. iberica* intercropped with chickpea (50% *L. iberica* + 50% chickpea), sole system of *L. iberica* (100%), and sole system of chickpea (100%); Autumn sowing date and spring sowing date; and measurements in the laboratory. Short interval (I_20_), Medium interval (I_40_), long interval (Is), Germination index (GP), Germination index (Gr), Vigor index (VI), Radicle length (Rl), and Plumule length (Pl), Thousand seed weight (TSW), Potassium (K), Stearic acid (SA), and oleic acid (OA).

The experiment was conducted using a split-factorial arrangement within a randomized complete block design (RCBD) with three biological replications. Each treatment combination was assigned to three independent field plots (biological replicates). The study comprised 18 treatment combinations resulting from three irrigation regimes (I_20_, I_40_, I_S_) × three cultivation systems (sole system: Ss of chickpea and *L. iberica*; intercropping: Ic) × two sowing dates (autumn: S₁; spring: S_2_). This resulted in a total of 54 experimental plots (18 treatments × 3 replications), with each plot measuring 5 m × 2 m (10 m²) containing eight sowing rows spaced 25 cm apart. The planting density was maintained at 40 plants m ⁻ ² for each species, resulting in 400 plants per plot (200 plants per species) in intercropping treatments. A schematic representation of the experimental setup, including irrigation regimes, cultivation systems, sowing dates, and laboratory measurements, is illustrated in Fig 1.

### Soil moisture determination and irrigation scheduling

Irrigation regime treatment was applied by extending irrigation intervals, thus causing a greater percentage of soil available water to be depleted before re-watering. Field capacity (Fc: 17.5%) and permanent wilting point (PWP: 6.5%) were empirically determined using soil cores (0–30 cm) via a pressure plate apparatus (Soil Moisture Equipment Corp. 1500, Soil Moisture Equipment Corp, USA) (at 33 kPa and 1.5 MPa, respectively) using Eq. (1).


MAD (%) = 100 × (Fc − θ)/ (Fc− PWP) (1)

Pre-irrigation soil moisture (θ) was measured gravimetrically every 48 h (post-irrigation) from root-zone samples (30 cm depth), oven-dried (Memmert UNB 500, Memmert, Germany) at 105 °C for 24 h (ASTM D2216) [32]. Therefore, treatments were irrigated when θ reached 15.3% (20% depletion) and 13.1% (40% depletion) of available water, respectively. Eq. (2).


In= (Fc−θ) × D× A)/100 (2)

In: Volume of water consumption for each plot, θ: Pre-irrigation soil moisture, D: Indicates the depth of root expansion, a: Experimental plot area (10 m²). Using the water meters (±2% accuracy) tracked applied volume per plot.

The irrigation treatments were applied at the beginning of plant establishment, specifically at the 8–12 leaf stage. The seed lots were left at room conditions to balance their moisture levels after being harvested between June 15–22 in both 2021–22 and 2022–23. The seeds were packed in aluminum foil bags (200 mm × 100 mm, L × W) and kept at 28 °C until used in experiments in November 2022 and 2023.

### Germination assays

Seed germination was evaluated in accordance with the International Seed Testing Association (ISTA, 2013) [33]. Standards Germination index (GI) was calculated according to ISTA rules, including final germination percentage (Gp) and germination rate (Gr) as components. Three replicates of 50 chickpea seeds and 50 *L. iberica* seeds were placed on filter paper in 90 mm diameter Petri dishes and moistened with 10 mL of water. A germination test was conducted over 14 days in an incubator (Binder KB 240, Binder, Germany) set at 10 °C, with a light/dark cycle of 16 h of light and 8 h of darkness, and maintained at 75% relative humidity. After this period, the normal seedlings were maintained at 65 °C for 2 days, after which their dry weight was measured using a scale (Sartorius CPA 1001, Sartorius, Germany) with an accuracy of 0.001 g. Thousand seed weight (TSW) was measured by weighing 1000 seeds in three replicates, following ISTA guidelines [33]. Seeds were randomly selected and weighed using the same precision scale. The Germin program was used to calculate the germination index (Gp, Gr). This program calculates D100, which represents the time required for germination to reach 100% of its maximum. Additionally, it computes the corresponding parameters for each plot by interpolating the germination increase curve over time [34]. The vigor index (VI) was calculated using the following Eq. (2) [3].


VI= germination index (Gp) ×SRL (3)

In which germination index (Gp) after 14 days and SRL is the seedling shoot and root length.

### Electrical conductivity (EC)

Following the International Seed Testing Association (ISTA, 2013) [33] guidelines, seed samples of chickpea and *L. iberica*—each weighing approximately 273.9 g and 2.75 g respectively, based on their average TSW—were soaked in 75 mL of deionized water at 25 °C for 24 h. Seeds with uniform size and weight were selected across all treatments to minimize physical variation. The EC of the resulting solution was measured using a calibrated conductivity meter and expressed as μS cm^-1^ g^-1^.

#### Biochemical determinations.

*Oil content:* A hexane solution n-hexane (ACS reagent grade, ≥ 99.9%, Sigma-Aldrich, Cat. No. 296090) was used to extract seed oil from ground seed samples in a Soxhlet apparatus (Merck Chemical Co, Germany, ACS grade, Reag. Ph. Eur., > = 99.9%). After filling the Soxhlet apparatus with solvent (150 mL), 10 g (W1) seeds from each treatment were added. Oil was extracted from brown seeds after boiling, evaporating, and condensing the solvent for 10 h. The samples were taken out of the Soxhlet after the requisite amount of time had passed, placed in the open air, and then again transported to an oven (Memmert UNB 500, Memmert, Germany) with a temperature of 80–100 °C for dehumidification. A desiccator was used to cool the samples after removing the moisture for 2 h. They were then weighed again after 35 min of cooling (W2). The oil percentage was calculated using the following formula: [35].


$$\text{Oil}\;\text{percentage}=\;\frac{\text{W1}-\text{W2}}{\text{W1}}\times \text{100}$$ (4)

*Fatty acids:* By methylating fixed oil into fatty acids and using gas chromatography (GC) (Agilent 7890A, Agilent Technologies, Inc. USA 2010), the fatty acid content of seeds was found. Oil samples were weighed and then treated with 3 mL of heptane solution (GC grade ≥99.9%, Sigma-Aldrich Cat. No. 246654) and 2 mL of sodium hydroxide solution (NaOH pellets, ≥ 98%, Merck, Cat. No. 106498) (0.01 M) with a shaker at 10000 rpm for 15 s in a 5 mL screw-top test tube. Finally, a microliter syringe was used to inject 1 μl of the FAME sample into the gas chromatograph. The analysis of the fatty acid methyl esters was performed using an Agilent 7890A GC (Agilent Technologies, Inc. USA 2010) fitted with a flame ionization detector (FID) and a BPX capillary (part number 054980) column (50 m, 0.22 mm internal diameter, 0.2 μmol film, nitrogen (99.9%, Air Products, Cat. No. NI UHP300) was the carrier gas with a head pressure of 4.136 bar, Agilent Technologies, Inc. 2010). For the first 10 minutes, the column’s initial temperature was held at 165 °C and then set to rise by 1.5 degrees Celsius every minute from 165 to 200 °C. Temperatures were set to 250 °C for the injector and 280 °C for the detector, respectively [36].

*Malondialdehyde (MDA):* For the analysis of MDA, 0.5 g of leaf material was homogenized using 5% trichloroacetic acid (TCA) (≥99.0% purity, Sigma-Aldrich, Cat. No. T6399), following the method of Hodges et al. (1999) slight modifications for estimating MDA [37]. The homogenates were then centrifuged at 10 g for 10 minutes using a centrifuge (MPW-351, MPW Med. Instruments, Poland), and the resulting supernatant was added to 20% TCA containing 0.5% thiobarbituric acid (TBA) (≥98% purity, Sigma-Aldrich, Cat. No. T5500). The mixture was incubated in a heater (Blockthermostat BT 200, Kleinfeld Labortechnik, Germany) at 95 °C for 30 minutes and then cooled on ice. The optical density was measured at 532 nm and 660 nm using a spectrophotometer (Lambda 25, Perkin Elmer, USA). The results were expressed in nmol per gram of fresh weight (nmol g^−1^ FW).

*Hydrogen peroxide content (H*_*2*_*O*_*2*_*):* For the determination of H_2_O_2_, 0.07 g of dry seeds was treated with 700 μL of 0.1% (w/v) trichloroacetic acid (TCA) (≥99.0% purity, Sigma-Aldrich, Cat. No. T6399) in an ice bath. The mixture was subsequently centrifuged at 15,000 g for 20 minutes at 4 °C. The supernatant was combined with 0.2 mL of phosphate buffer (10 mM, pH 7) and 1 mL of potassium iodide (1 M) (≥99.5% purity, Sigma-Aldrich, Cat. No. P8286). After incubating for 1 h at room temperature (25 °C) in the dark, the absorbance was measured at 390 nm. A standard curve was used for the analysis of H_2_O_2_ [38].

*Seed mucilage:* To measure the mucilage content of the seeds, 10 g of seeds were placed in boiling water at 100 °C for 30 min. After the extraction period, the extract was allowed to cool to room temperature. Then, the extract was filtered through glass wool, and the volume of the filtrate was reduced using rotary evaporation. Next, ethanol 96% (v/v), ACS reagent grade (≥99.8%, Sigma-Aldrich, Cat. No. 459836) was added to the extract to achieve a final concentration of 80% (v/v), causing the mucilage to precipitate. After 24 hours at 25 °C, the precipitate was removed by centrifugation (4500 g for 30 min at 5 °C) and homogenized in water. Finally, the supernatant was poured off, and the beaker containing the precipitate was dried in an oven (Memmert UNB 500, Memmert, Germany) maintained at 50 °C. The weight of the dry precipitate was used as the measure of the total mucilage content [39].

*Absorption of nutrients:* Filtered sample by grinding and ashing 1 gram of dry matter for 24 hours at 50–600 °C (white color) and then digestion with 100 mL volumetric flasks of 0.2 M hydrochloric acid (HCL) (37%, TraceSELECT Ultra, Sigma-Aldrich, Cat. No. 84415) for 30 minutes in a water bath (Julabo SW23, ± 0.1 °C accuracy) was done. The samples were diluted with 100 mL of distilled water after filtering with filter paper (Whatman No. 42).

To measure nitrogen (N), 25 mL of the filtered sample was poured into the balloon of the device and distilled using potassium hydride (40% w/v, ACS grade, Sigma-Aldrich, Cat. No. 221473) and 0.2% boric acid (4% w/v, ACS grade, Sigma-Aldrich, Cat. No. B6768), and the released ammonia was then removed by distillation and collected by boric acid. Using 0.01 hydrochloric acid and normal acid, the resulting solution was also titrated. Following the titration stage, N was evaluated using Kjeldahl (Büchi Labortechnik, Switzerland) [40].

The seed phosphorus (P) concentration is determined by heating 2 mL of the filtrated sample in a bain-marie (Memmert WNB 14, 95 °C ± 1 °C) for 30 minutes with 10 mL ammonium molybdate (1% in 0.5 M H₂SO₄, Sigma-Aldrich, Cat. No. 277908) and 2 mL of ascorbic acid (5% w/v, ACS grade, Sigma-Aldrich, Cat. No. A5960). In the end, a spectrophotometer (Lambda 25, Perkin Elmer, USA) was used to measure the concentration of P at 700 nm [41].

To measure potassium (K) [42], 1 mL of the filtered sample was diluted with 9 mL of cesium chloride 1% (Sigma-Aldrich, Cat. No. 20994), and the absorbance was measured with a film photometer (BWB XP, BWB Technologies, UK) at a wavelength of 766.5 nm.

*Statistical analysis:* The SAS was used to analyze the data (SAS version 9.2, SAS Institute, Cary, NC, USA). A combined analysis of variance (ANOVA) was conducted, and the LSMEANS test was used to compare means at *P ≤ *0.05. Graphs were created in Excel. Heat map and Pearson correlations were carried out using the R packages ‘FactoMinerR’ [43] and ‘factextra’ [44].

## Results

### Impact of intercropping and drought stress on germination enhancement across sowing dates

**Fig 2:**
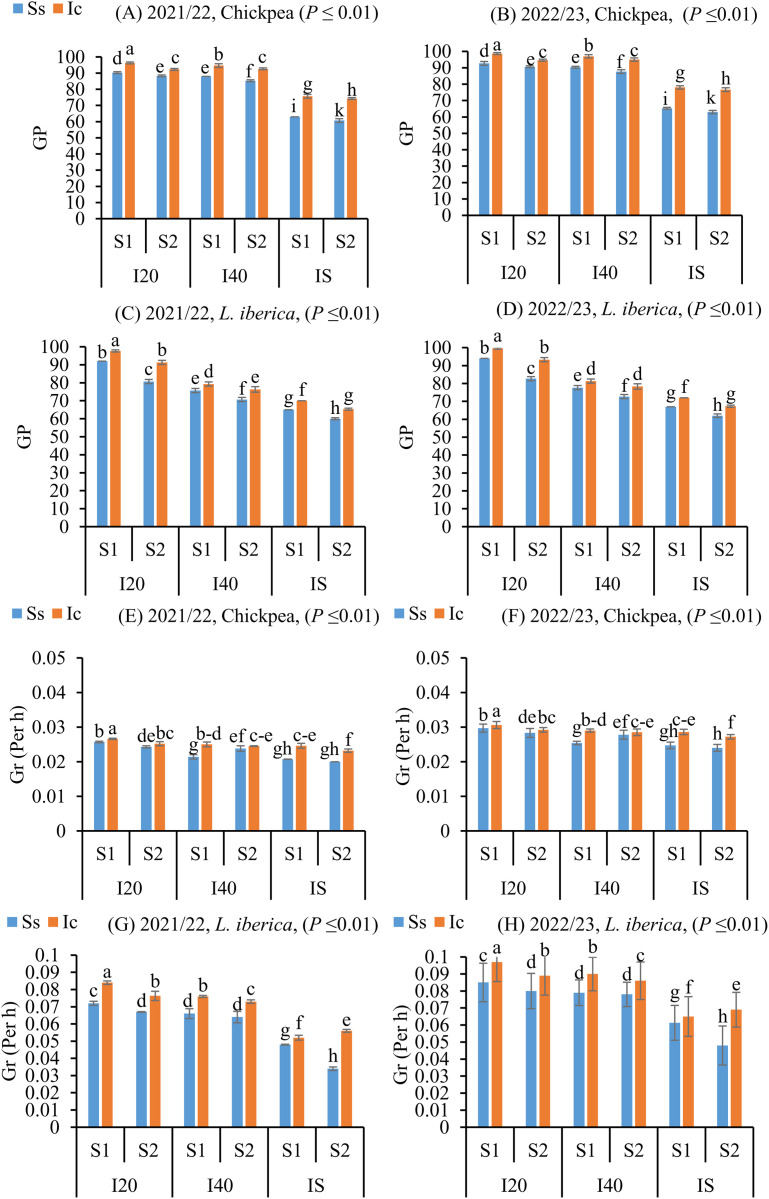
Germination index (GP), and Germination index (Gr) of chickpea (A, B, E, and F), and *L. iberica* (C, D, G, and H) during the growing seasons of 2021/22 and 2022/23. Sole system (Ss), Intercropping (Ic) of 50% chickpea: 50% *L. iberica*, Autumn sowing date (S_1_), Spring sowing date (S_2_), Short interval (I_20_), Medium interval (I_40_), long interval (Is). Error bars indicate standard deviation (SD) (n = 3). LSMEANS within each column of each section followed by the same letter are not significantly different (*P ≤ *0.05).

Seed quality and physiological and biochemical properties are important for the proper growth and development of the crop and also to have a higher yield. So, the ANOVA showed significant effects of irrigation regime, cultivation system, and sowing date on germination indexs, physiological, physical, and biochemical traits (S2, S3, S5, S6 Tables), and oil and fatty acid compositions (S4 Table). An increase in germination index (Gp) (Fig 2A, B, C, and D) (*P* ≤ 0.01), Gr (Fig 2E, F, G, and H) (*P* ≤ 0.01), Rl (S2 Fig in S1 File. A, B, C, and D) (*P* ≤ 0.01), Pl (S2 Fig in S1 File. E, F, G, and H) (*P* ≤ 0.01), seedling dry weight (S3 Fig in S1 File. A, B, C, and D) (*P* ≤ 0.01), VI (S3 Fig in S1 File. E, F, G, and H) (*P* ≤ 0.01), and TSW (S4 Fig in S1 File. A, B, C, and D) (*P* ≤ 0.05, *P* ≤ 0.01) was observed in chickpea and *L. iberica* seeds matured under the Ic (I_20_S_1_)> Ic (I_40_S_1_)> Ic (I_S_S_1_) treatments compared with those increased under other treatments. In the intercropping treatments, the germination index (Gp), VI, seedling dry weight, germination index (Gr), RL, and Pl were more than in other sole system treatments in both chickpea and *L. iberica* species. The germination indexs under Ss (I_S_S_2_) treatment was much lower than in other treatments in chickpea and *L. iberica*, with chickpea seeds was more than that in *L. iberica*. In addition, the seeds subjected to spring sowing date high temperature (S_1_) and increasing irrigation intervals (Is) in the field declined significantly in germination index (Gp, Gr) after reaching physiological maturity (Fig 2A–H). In contrast, in all sole system treatments, the spring sowing date could not mitigate the adverse effects of water deficit on *L. iberica* and chickpea germination indexs. This was due to the fact that no proper development of seeds resulted in less accumulation of food reserves and recorded lower seed weight, which ultimately might have resulted in lower germination.

### Enhancement of seed mucilage in L. iberica under intercropping and autumn sowing

**Fig 3:**
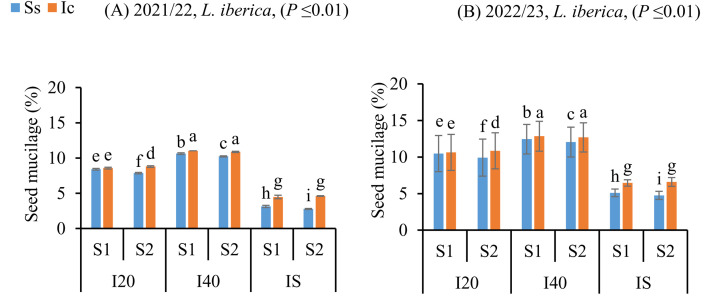
Seed mucilage content of *L. iberica* (A, B) during the growing seasons of 2021/22 and 2022/23. Sole system (Ss), Intercropping (Ic) of 50% chickpea: 50% *L. iberica*, Autumn sowing date (S_1_), Spring sowing date (S_2_), Short interval (I_20_), Medium interval (I_40_), long interval (Is). Error bars indicate standard deviation (SD) (n = 3). LSMEANS within each column of each section followed by the same letter are not significantly different (*P ≤ *0.05).

The adaptive value of mucilage has attracted the attention of plant ecologists, and various possible functions of mucilage have been proposed in the literature [45–48]. One commonly discussed ecological adaptation of seed mucilage is its ability to facilitate water absorption and retain moisture for plants that thrive in water-deficient conditions found in arid and semiarid environments [49]. The maximum and minimum mucilage content of *L. iberica* was obtained from the Ic (I_40_S_1_) treatment and the Ss (I_S_S_1_) treatment, respectively (*P* ≤ 0.01) (Fig 3A, B). Mucilage content of *L. iberica* seeds was higher than that of the sole system across all intercropping treatments. In addition, among sowing dates, the spring sowing date (S_2_) significantly reduced the seed mucilage, while the autumn sowing date (S_1_) produced the maximum seed mucilage.

### MDA content response in Chickpea and L. iberica seeds to intercropping, sowing dates, and drought stress

**Fig 4:**
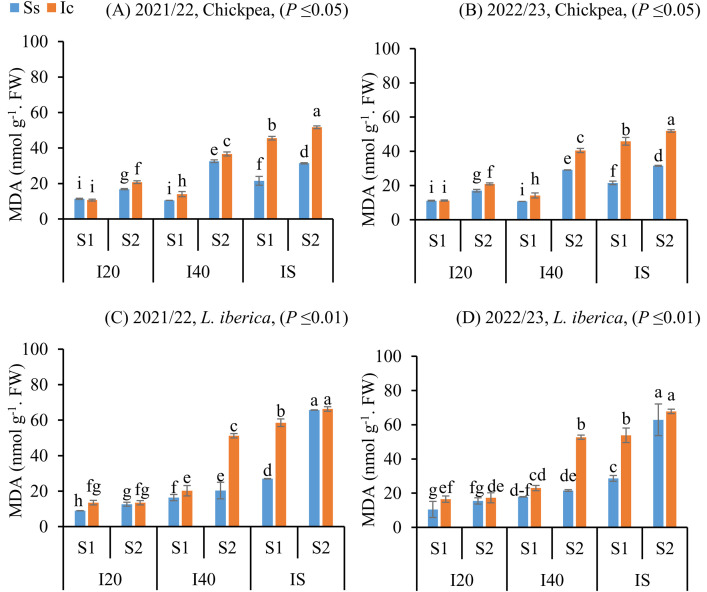
Malondialdehyde (MDA) of chickpea (A, B), and *L. iberica* (C, D) during the growing seasons of 2021/22 and 2022/23. Sole system (Ss), Intercropping (Ic) of 50% chickpea: 50% *L. iberica*, Autumn sowing date (S_1_), Spring sowing date (S_2_), Short interval (I_20_), Medium interval (I_40_), long interval (Is). Error bars indicate standard deviation (SD) (n = 3). LSMEANS within each column of each section followed by the same letter are not significantly different (*P ≤ *0.05).

Intercropping, sowing date, and irrigation interval significantly affected the MDA contents of seeds matured of both chickpea and *L. iberica* species during both study years. Intercropping treatments increased the MDA contents of seeds matured and stored of chickpea (*P* ≤ 0.05) and *L. iberica* (*P* ≤ 0.01) species, except in the sole system, where a decrease was noted for MDA contents during both study years (Fig 4). With the increasing irrigation interval, the spring sowing date in the intercropping treatment could alleviate the detrimental effects of water stress by increasing the MDA contents of seeds matured of chickpea (Fig 4A, B) and *L. iberica* (Fig 4C, D). The Ic (I_S_S_2_) treatment increased the MDA contents compared with other treatments, but there was no significant difference (*P* ≤ 0.01) in the MDA content of seeds matured of *L. iberica* for Ic (I_S_S_1_), Ss (I_S_S_1_), Ic (I_S_S_2_), and Ss (I_S_S_2_) treatments (Fig 4C, D).

### *H*_*2*_*O*_*2*_ *content response in Chickpea and L. iberica seeds to intercropping and sowing dates under drought stress*

**Fig 5:**
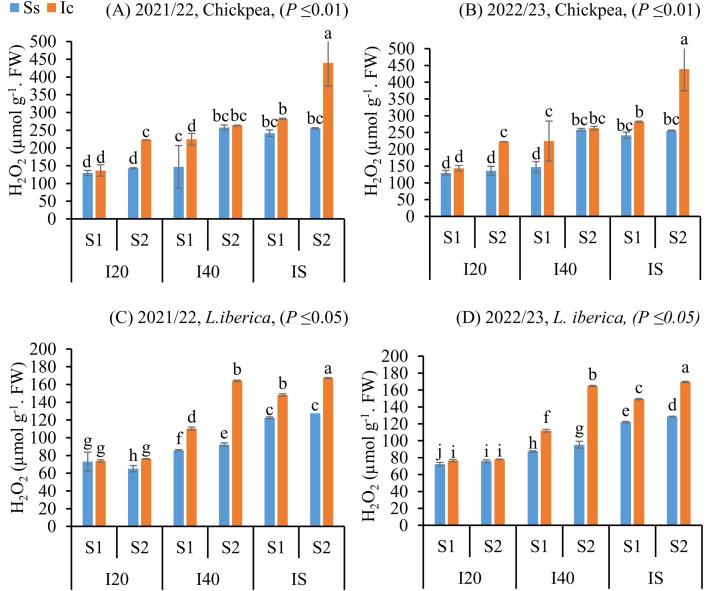
Hydrogen peroxide content (H_2_O_2_) of chickpea (A, B), and *L. iberica* (C, D) during the growing seasons of 2021/22 and 2022/23. Sole system (Ss), Intercropping (Ic) of 50% chickpea: 50% *L. iberica*, Autumn sowing date (S_1_), Spring sowing date (S_2_), Short interval (I_20_), Medium interval (I_40_), long interval (Is). Error bars indicate standard deviation (SD) (n = 3). LSMEANS within each column of each section followed by the same letter are not significantly different (*P ≤ *0.05).

In response to the increasing irrigation intervals during the spring sowing of matured seeds of chickpea (**P* *≤ 0.01) and *L. iberica* (*P* ≤ 0.05), aimed at avoiding water deficits in the late season, H_2_O_2_ activity significantly increased in the Ic (I_S_S_2_) treatment (Fig 5). Intercropping increased H_2_O_2_ contents of seeds matured of chickpea and *L. iberica*, except in the sole system, where a decrease was noted for H_2_O_2_ contents during both study years (Fig 5). Despite the observed increase in H_2_O_2_ content under Ic (I_S_S_1_) and Ic (I_S_S_2_) treatments, this elevation is likely indicative of adaptive oxidative signaling rather than stress-induced cellular damage, reflecting intercropping-mediated enhancement in drought resilience [50].

### Intercropping mitigates seed EC in Chickpea and L. iberica under water stress

**Fig 6:**
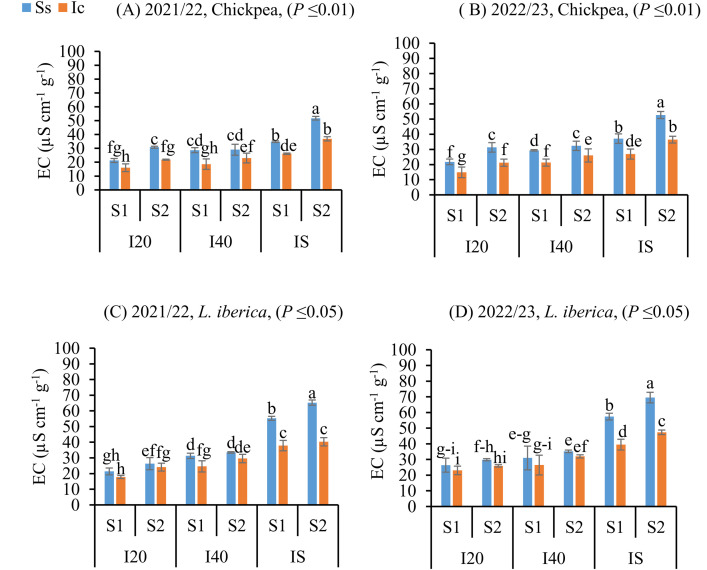
Electrical conductivity (EC) of chickpea (A, B), and *L. iberica* (C, D) during the growing seasons of 2021/22 and 2022/23. Sole system (Ss), Intercropping (Ic) of 50% chickpea: 50% *L. iberica*, Autumn sowing date (S_1_), Spring sowing date (S_2_), Short interval (I_20_), Medium interval (I_40_), long interval (Is). Error bars indicate standard deviation (SD) (n = 3). LSMEANS within each column of each section followed by the same letter are not significantly different (*P ≤ *0.05).

With increasing irrigation interval from I_20_ to IS, chickpea (**P* *≤ 0.01) and *L. iberica* (**P* *≤ 0.05) EC of seeds matured increased in Ic (I_S_S_2_) treatment during both study years, but the most EC was observed in seeds matured of chickpea and *L. iberica* species under the Ss (I_S_S_2_) treatment (Fig 6). Compared with other treatments, in both chickpea and *L. iberica* species, a decrease in EC was observed in Ic (I_20_S_2_) treatment that was no significant difference (*P *≤ 0.05) in EC of seeds matured of *L. iberica* for Ic (I_20_S_1_), Ss (I_20_S_1_), Ic (I_20_S_2_), and Ss (I_20_S_2_) treatments. The highest EC values were found in the seeds matured of *L. iberica* (Fig 6C, D). Higher EC was noticed in the sole system compared to intercropping because of slightly low germination, less seed viability, high frazzle that increases the membrane damage, disturbance of enzyme activity, and other cell structures [51]. They reported that high frazzle increases membrane damage and disturbance of certain enzyme activity responsible for the degradation of macromolecules into micromolecules within the seed and other cell structures.

### N-P-K concentration reduction under drought stress and the modulatory role of autumn sowing and intercropping

**Fig 7:**
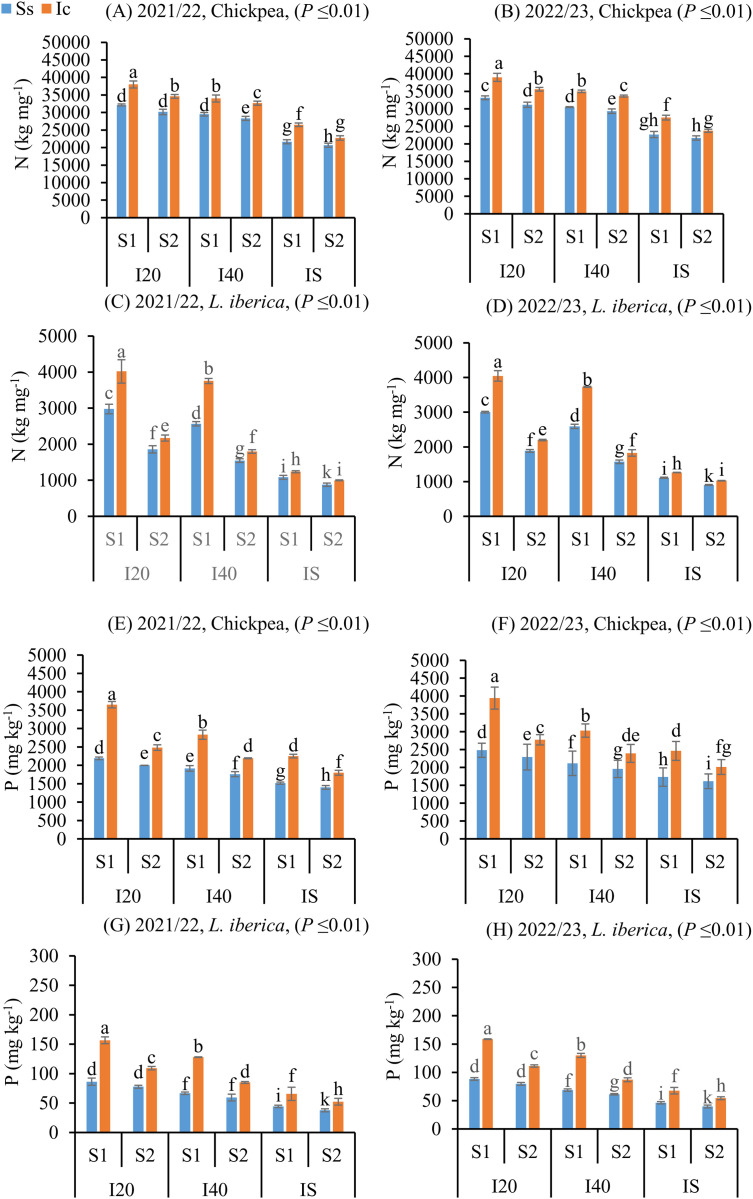
Nitrogen (N) and Phosphorus (P) of chickpea (A, B, E, and F), and *L. iberica* (C, D, G, and H) during the growing seasons of 2021/22 and 2022/23. Sole system (Ss), Intercropping (Ic) of 50% chickpea: 50% *L. iberica*, Autumn sowing date (S_1_), Spring sowing date (S_2_), Short interval (I_20_), Medium interval (I_40_), long interval (Is). Error bars indicate standard deviation (SD) (n = 3). LSMEANS within each column of each section followed by the same letter are not significantly different (*P ≤ *0.05).

The concentration of P, N, (**P* *≤ 0.01) (Fig 7A–H) and K (*P *≤ 0.01) (S5 Fig in S1 File. A, B, C, and D) in chickpea and *L. iberica* seeds matured decreased as the irrigation interval increased from I_20_ to IS. Thus, in all treatments during the two years of study, the concentration of in P, N, and K in the Ic (I_20_S_1_) treatment was significantly higher than in other treatments. Furthermore, a significant increase P, N, and K concentration in both species of chickpea and *L. iberica* on the autumn sowing date indicated that P, N, and K uptake was enhanced with a decrease in temperature and an increase in rainfall. Compared with *L. iberica*, seeds matured of chickpea contained higher P, N, and K concentrations regardless of sowing date or irrigation treatment, suggesting this species’ efficacy in uptaking soluble P, N, and K from the soil.

### Enhancement of oil quality and fatty acid profiles in L. iberica under intercropping and autumn sowing

**Fig 8:**
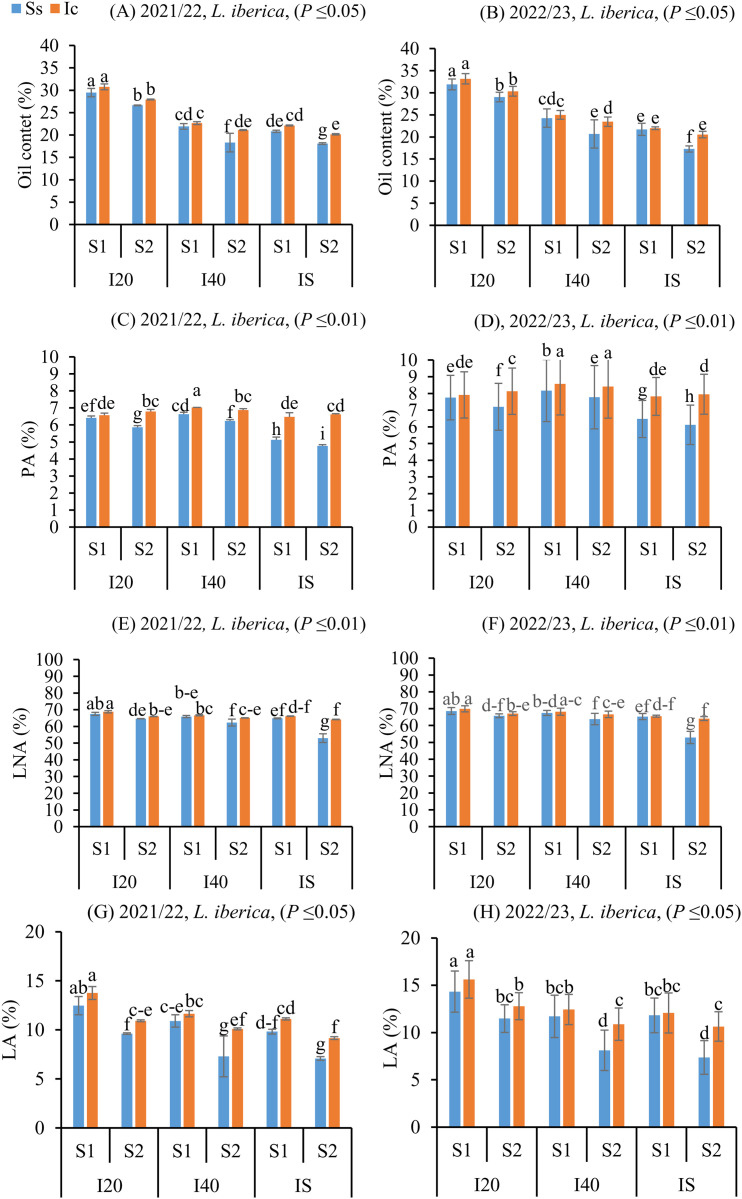
Oil content, Palmitic acid (PA), Linolenic acid (LNA), Linoleic acid (LA) of *L. iberica* (A-H) during the growing seasons of 2021/22 and 2022/23. Sole system (Ss), Intercropping (Ic) of 50% chickpea: 50% *L. iberica*, Autumn sowing date (S_1_), Spring sowing date (S_2_), Short interval (I_20_), Medium interval (I_40_), long interval (I_S_). Error bars indicate standard deviation (SD) (n = 3). LSMEANS within each column of each section followed by the same letter are not significantly different (*P ≤ *0.05).

Although the oil content in *L. iberica* decreased with longer irrigation intervals (**P* *≤ 0.05) (Fig 8A, B), the autumn sowing date significantly increased the oil content of the matured seeds compared to the spring sowing date. The oil content of seeds matured in *L. iberica* was consistently higher than that of the sole system across all irrigation regimes and intercropping treatments. Fig 8 shows that increasing irrigation intervals led to notable changes in the fatty acid compositions of matured seeds in *L. iberica*. Specifically, as irrigation intervals increased from I_20_ to I_40_, the levels of fatty acids such as oleic acid (OA) (**P* *≤ 0.01) (S6 Fig in S1 File. C, D), stearic acid (SA) (**P* *≤ 0.01) (S6 Fig in S1 File. A, B), and palmitic acid (PA) (**P* *≤ 0.01) (Fig 8C, D) initially rose, then declined with further increases in irrigation intervals under the I_S_ treatment. In contrast, the levels of linoleic acid (LA) (**P* *≤ 0.05) (Fig 8G, H) and linolenic acid (LNA) (**P* *≤ 0.01) (Fig 8E, F) decreased. The results indicate that intercropping and the autumn sowing date significantly enhanced the oil quality of matured seeds in *L. iberica* by increasing fatty acid content. Among all irrigation regimes, cultivation systems, and sowing dates, the highest levels of LNA and LA were observed in the Ic (I_20_S_1_) treatment, while the highest levels of OA, SA, and PA were found in the Ic (I_40_S_1_) treatment (Fig 8A–L).

### Multivariate correlation and cluster analysis of physiological-biochemical traits in Chickpea and L. iberica

**Fig 9:**
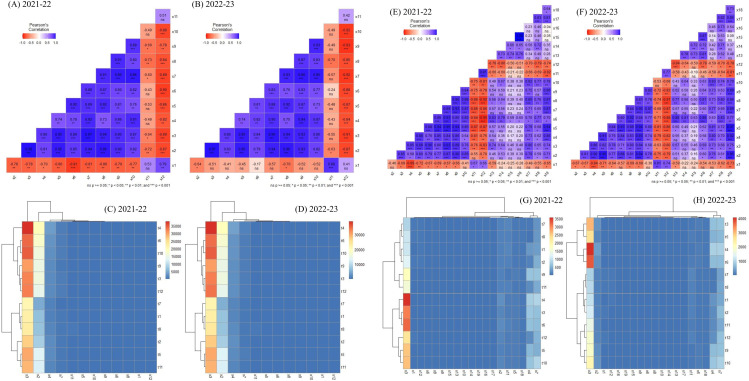
Heat map of Pearson correlations (A, B, E, and F), and dendrogram clustering (C, D, G, and H) among studied traits in chickpea and *L. iberica* species, X1: Malondialdehyde (MDA), X2: Potassium (K), X3: Nitrogen (N), X4: Phosphorus (P), X5: germination index (Gp), X6: germination index (Gr), X7: vigor index (VI), X8: Seedling dry weight, X9: Plumule length (Pl), X10: Radicle length (Rl), X11: Hydrogen peroxidase, X12: Electrical conductivity (EC), t1: Ss (I_S_S_1_), t2: Ic (I_S_S_1_), t3: Ss (I_20_S_1_), t4: Ic (I_20_S_1_), t5: Ss (I_40_S_1_), t6: Ic (I_40_S_1_), t7: Ss (I_S_S_2_), t8: Ic (I_S_S_2_), t9: Ss (I_20_S_2_), t10: Ic (I_20_S_2_), t11: Ss (I_40_S_2_), t12: Ic (I_40_S_2_).

During two years of study on the chickpea plant, the correlation heat map exhibited a positive relationship among K, N, P, germination index (Gp), germination index (Gr), VI, seedling dry weight, Pl, and Rl. Also, a positive correlation was observed among MDA and EC. Finally, the results determined a significant negative correlation between the two groups mentioned above (Fig 9A, B). The dendrogram clustering heat map analysis showed that the evaluated traits were classified into two clusters; cluster 1 contained K, P, VI, H_2_O_2_, MDA, EC, germination index (Gp), Pl, seedling dry weight, and Rl, and cluster 2 consisted of N. On the other hand, the chickpea plant subjected to these experimental treatments revealed two groups, as group 1 included the Ss (I_20_S_1_), Ic (I_20_S_1_), Ic (I_20_S_2_), Ss (I_20_S_2_), Ic (I_40_S_1_), and Ic (I_40_S_2_) treatments. Group 2 had the Ss (I_S_S_1_), Ss (I_S_S_2_), Ic (I_S_S_1_), Ic (I_S_S_2_), Ss (I_40_S_1_), and Ss (I_40_S_2_) treatments (Fig 9C, D).

In addition, in the *L. iberica* plant, the correlation heat map exhibited a positive relationship among K, N, P, germination index (Gp), germination index (Gr), VI, seedling dry weight, Pl, Rl, LNA, LA, and oil content. While, seed mucilage content, PA, SA, OA, N, germination index (Gr), seedling dry weight, and Rl had a strong positive correlation with each other (Fig 9E, F). Also, there was a positive correlation among MDA, EC, and H_2_O_2_. The dendrogram clustering heat map analysis showed that the evaluated traits were classified into two clusters; cluster 1 contained K, P, germination index (Gp), germination index (Gr), VI, seedling dry weight, Pl, Rl, seed mucilage content, MDA, EC, H_2_O_2_, OA, LNA, LA, PA, SA, and oil content, while cluster 2 consisted of N. On the other hand, the chickpea plant subjected to these experimental treatments revealed two groups, with group 1 including the Ss (I_S_S_2_), Ic (I_S_S_2_), Ss (I_S_S_1_), Ic (I_S_S_1_), Ss (I_20_S_2_), and Ss (I_40_S_2_) treatments. Group 2 had the Ic (I_20_S_1_), Ss (I_20_S_1_), Ic (I_40_S_1_), Ic (I_40_S_2_), Ss (I_40_S_1_), and Ss (I_20_S_2_) treatments (Fig 9G, H).

## Discussion

The observed improvement in germination indexs—including germination percentage (Gp), radicle length (Rl), plumule length (Pl), seedling dry weight, and vigor index (VI)—in mature seeds of chickpea and *L. iberica* under intercropping treatments Ic (I_20_S_1_) and Ic (I_40_S_1_) can be attributed to enhanced maternal growth conditions. Such improvements are likely driven by facilitative resource partitioning within intercropping systems, where complementary root architectures (deep-rooting chickpea vs. shallow-rooting *L. iberica*) reduce competition and alter microclimatic conditions via canopy shading [52–54]. These favorable maternal conditions not only improve seedling traits but also influence seed viability and longevity, prompting further consideration of underlying physiological mechanisms. Notably, TSW also increased under the same intercropping treatments, mirroring the trends observed in germination indices. This suggests that improved maternal conditions positively influenced both physiological and physical seed quality traits.

In particular, seed longevity under intercropping appears to reflect reduced oxidative stress and more balanced soil interactions, unlike sole systems that showed substantial declines in germination metrics. These declines were accompanied by elevated levels of H_2_O_2_ and MDA, indicating excessive ROS accumulation [55–57]. Interestingly, intercropping treatments such as Ic (I_S_S_1_) and Ic (I_S_S_2_) also exhibited moderate increases in H_2_O_2_, which may function as signaling molecules to activate antioxidant defenses. This dual role of ROS—as both stress agents and signaling mediators—highlights the complexity of plant responses under intercropping conditions [58,59].

Such responses also vary across species. *L. iberica* seeds consistently accumulated higher levels of H_2_O_2_ and MDA than chickpea, correlating with its elevated content of unsaturated fatty acids, which are more prone to peroxidation [60,61]. The oxidative damage is further intensified by lipoxygenase activity, which catalyzes the breakdown of unsaturated fats into reactive molecules [62]. Despite this vulnerability, intercropping treatments—particularly Ic (I_20_S_1_), Ic (I_40_S_1_), and Ic (I_S_S_2_)—promoted the accumulation of both saturated and unsaturated fatty acids in *L. iberica*. These improvements may be attributed to chickpea-derived phenolics, enhanced rhizosphere moisture, and reduced lipid oxidation [27,63,64]. Building on this, recent studies suggest that stress-induced modulation of triacylglycerol metabolism can shape fatty acid profiles, adding another layer of biochemical regulation [65].

Improved seed lipid profiles are closely linked to nutrient dynamics, which in intercropping systems benefit from root-level complementarity. Chickpea contributes biologically fixed nitrogen via rhizobial symbiosis (up to 85%), while *L. iberica* mobilizes P through citrate/malate exudation [66–68]. Together, these root functions enhance nutrient acquisition and seed reserve accumulation, supporting robust embryo development. These reserves—rich in amino acids and nucleotides—enable sustained heterotrophic growth and metabolic activity during early seedling establishment [69,70], suggesting a direct link between maternal nutrient provisioning and seed vigor.

This link is further supported by enzymatic activity within germinating seeds. Nutrient-rich seeds showed elevated α-amylase and protease levels, facilitating reserve mobilization, while enhanced catalase, superoxide dismutase, and peroxidase activities mitigated ROS damage during early growth [71–73]. These protective mechanisms complement the rhizosphere interactions observed during intercropping, where differential root foraging and microbial associations improve P solubilization and AMF colonization [74–76]. Evidence from chickpea–flax intercropping supports this, showing a 30% increase in P uptake via rhizosphere acidification [77]. Moreover, root niche differentiation mitigated water stress through reduced interspecific competition, aligning with findings from Duchene et al. [30].

Such resource optimization is especially valuable under drought stress. AMF-mediated nutrient transfer has been shown to improve P availability by 25%, and quantified citrate/malate exudation from *L. iberica* roots (2.1 µmol/g) supports enhanced rhizosphere activation [78–80]. These processes not only promote nutrient uptake but also contribute to soil moisture retention and improved diffusion kinetics—reinforcing the protective benefits of intercropping under environmental constraints. These processes not only promote nutrient uptake but also contribute to soil moisture retention and improved diffusion kinetics—reinforcing the protective benefits of intercropping under environmental constraints.

The impact of water scarcity was particularly evident in sole systems, where reduced soil hydration limited photosynthetic efficiency and assimilate allocation, ultimately lowering seedling biomass [49,50]. Conversely, intercropping moderated microclimatic conditions; shading by chickpea canopy reduced soil temperature and evaporation, supporting hydration during seed maturation in *L. iberica* [30,81]. These modifications to vapor pressure and moisture retention reinforce the role of canopy architecture in sustaining seed quality under stress.

Sowing date and seasonal timing also influenced seed performance. Delayed sowing and shortened vegetative growth periods diminished germination index, as confirmed by Haro et al. (2007), who showed that seed harvesting time and growth duration are critical determinants of seed quality [82]. Higher temperatures during spring sowing abbreviated vegetative stages, limiting nutrient acquisition [83]. Yet, intercropping offset these effects through temporal niche differentiation—chickpea thriving in cooler seasons while *L. iberica* flourished in warmer conditions. This staggered phenology reduced competition and enhanced seasonal resource capture [84,85].

Altogether, this study provides valuable insights into intercropping effects on seed quality, several limitations should be acknowledged. First, our focus on biochemical markers (MDA, H_2_O_2_, fatty acids) and germination indexs did not encompass physical seed quality parameters such as seed size or purity and storage stability, which are important for commercial seed standards. Second, the two-year study duration limits our ability to assess long-term climate variability impacts on seed quality trends. Additionally, the experiment was conducted at a single geographical location, which may affect the generalizability of results to other arid/semi-arid regions. While these constraints are inherent to controlled physiological studies, they do not invalidate our core findings regarding the stress-mitigating mechanisms of intercropping. Future research should address these gaps through multi-location field trials incorporating comprehensive seed quality assessments (physical, biochemical, and physiological) over extended growing seasons, while advanced root-soil-microbe investigations (e.g., imaging/sequencing) could further optimize system adaptability across environmental extremes.

## Conclusion

This study demonstrates that chickpea-*L. iberica* intercropping effectively enhances drought resilience in seed production systems through threshold-dependent mechanisms. The superior performance of Ic (I_20_S₁) and Ic (I_40_S₁) treatments, achieving germination rates exceeding 85%, results from complementary biochemical and ecological interactions between the two species. Chickpea protective root exudates combine with optimized resource use and moderated microclimates to sustain seed quality under water-limited conditions. Intercropping enhances drought resilience under I_20_ and I_40_ through these synergistic effects, though the elevated H_2_O_2_ levels observed in Ic (I_S_S₁/I_S_S_2_) treatments reveal a stress-intensity limit – under I_S_, proximity-induced root competition partially offsets benefits by triggering oxidative markers, while still maintaining seed quality superior to sole systems. These findings offer immediately applicable solutions for sustainable agriculture in arid regions. The study establishes that intercropping chickpea with *L. iberica* provides a reliable strategy for stabilizing seed production in drought-prone agricultural systems through complementary stress-resilience mechanisms.

## Supporting information

S1 File Germination assays and biochemical analyses of chickpea and *L. iberica* seeds according to the maternal plant environment. (ZIP)

S2 File All Raw Data available in Standard Format. (XLSX)

S1 Table Meteorological data recorded at the experimental site from November to June during the 2021–22 and 2022–23 growing seasons. (DOCX)

S2 Table The combined analysis of variance for the effect of the maternal environment conditions on germination assays and biochemical determinations of *L. iberica* in 2021−22 and 2022−23. (DOCX)

S3 Table The combined analysis of variance for the effect of the maternal environment conditions on absorption of nutrients of *L. iberica* in 2021−22 and 2022−23. (DOCX)

S4 Table The combined analysis of variance for the effect of the maternal environment conditions on oil content and fatty acids of *L. iberica* in 2021−22 and 2022−23. (DOCX)

S5 Table The combined analysis of variance for the effect of the maternal environment conditions on germination assays and biochemical determinations of chickpea in 2021−22 and 2022−23. (DOCX)

S6 Table The combined analysis of variance for the effect of the maternal environment conditions on absorption of nutrients of chickpea in 2021−22 and 2022−23. (DOCX)
