# Supplementary figures and images for: Seed quality as affected by intercropping of Chickpea and L. iberica
Source: PLoS One. 2025 Oct 30;20(10):e0332264. doi: 10.1371/journal.pone.0332264 (PMC12574852; doi:10.1371/journal.pone.0332264)

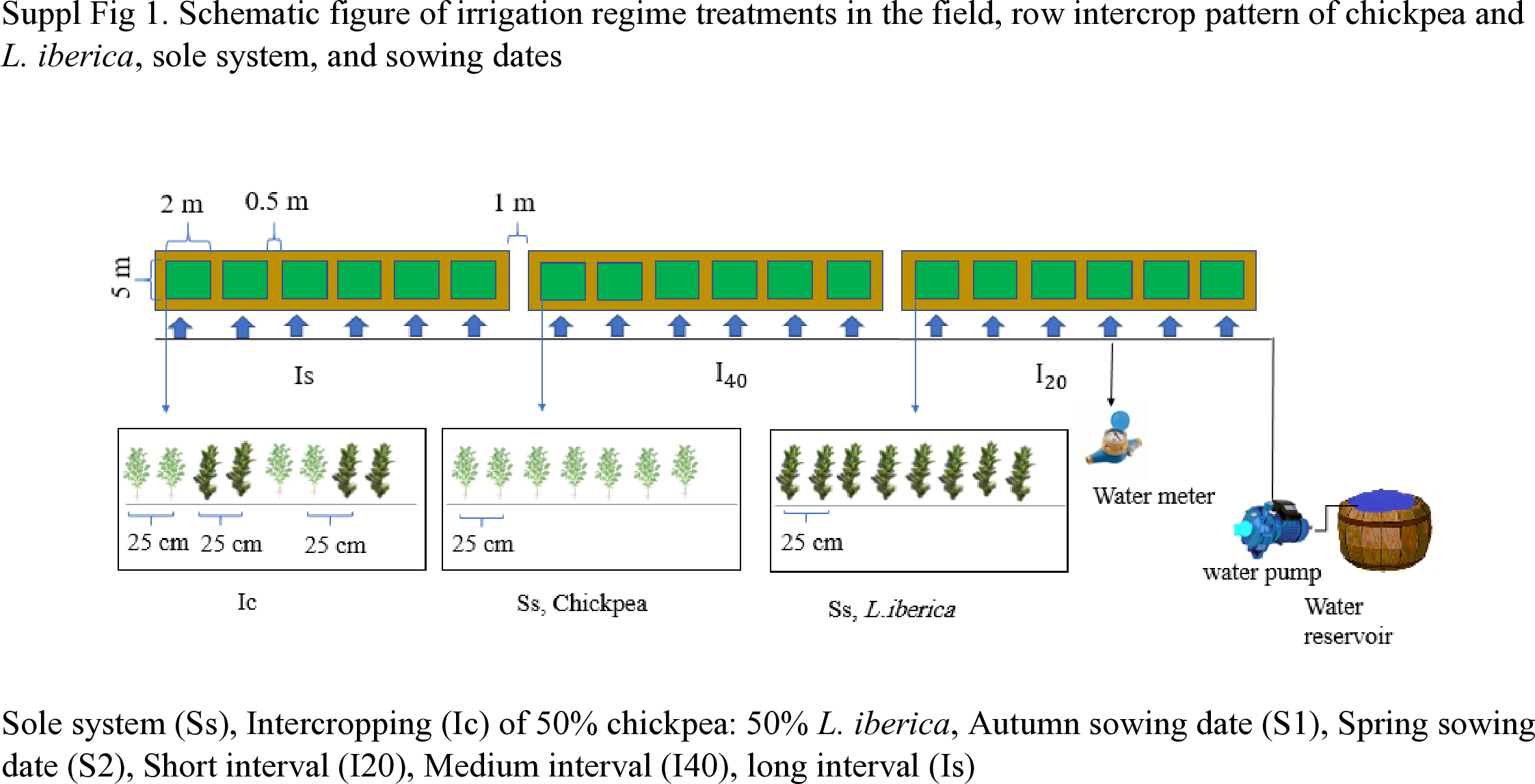

Supplement: S1 File — (ZIP) [file pone.0332264.s001.zip › PACE Corrected/S1 File.tif]

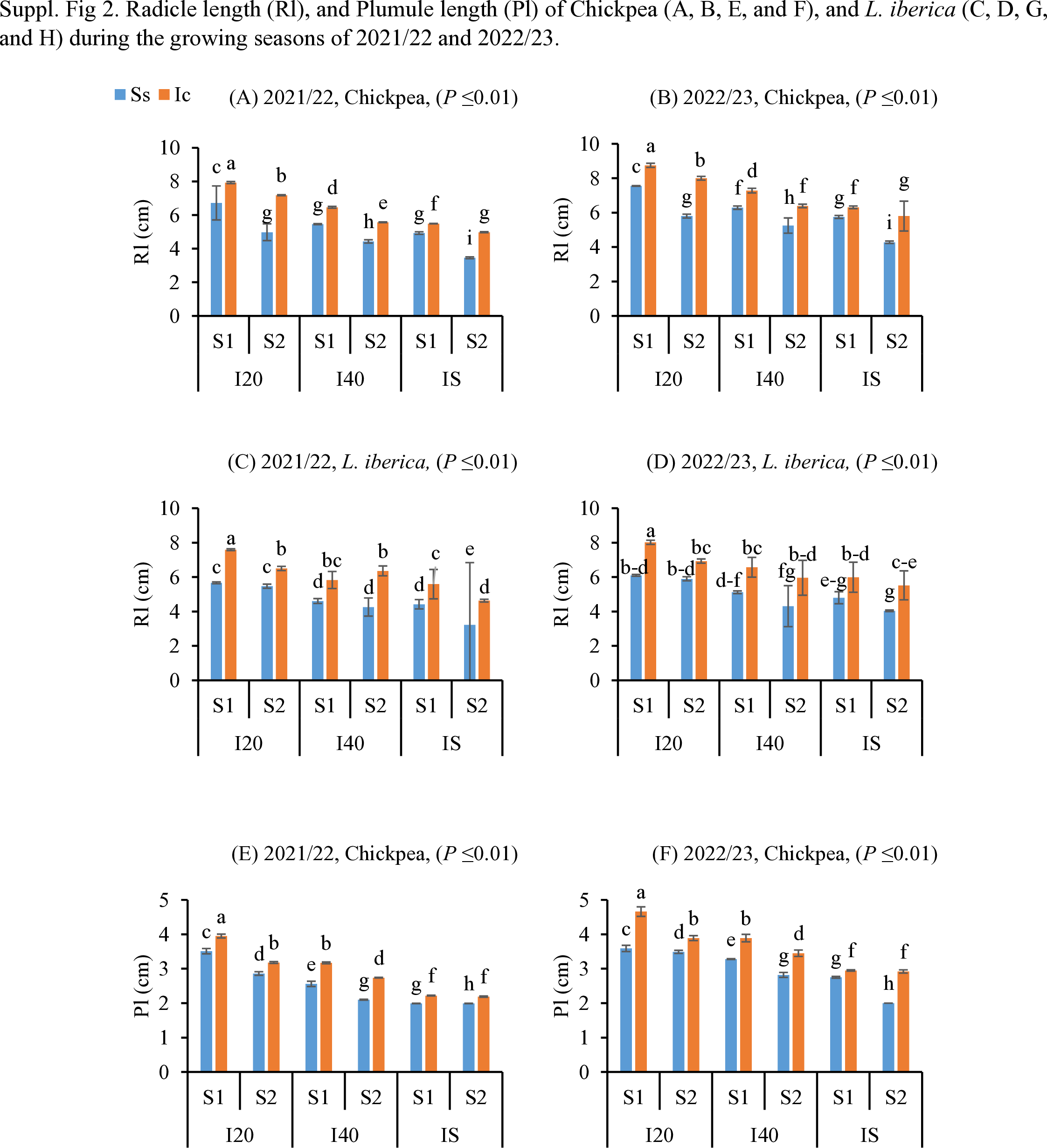

Supplement: S1 File — (ZIP) [file pone.0332264.s001.zip › PACE Corrected/S1 File.tif]

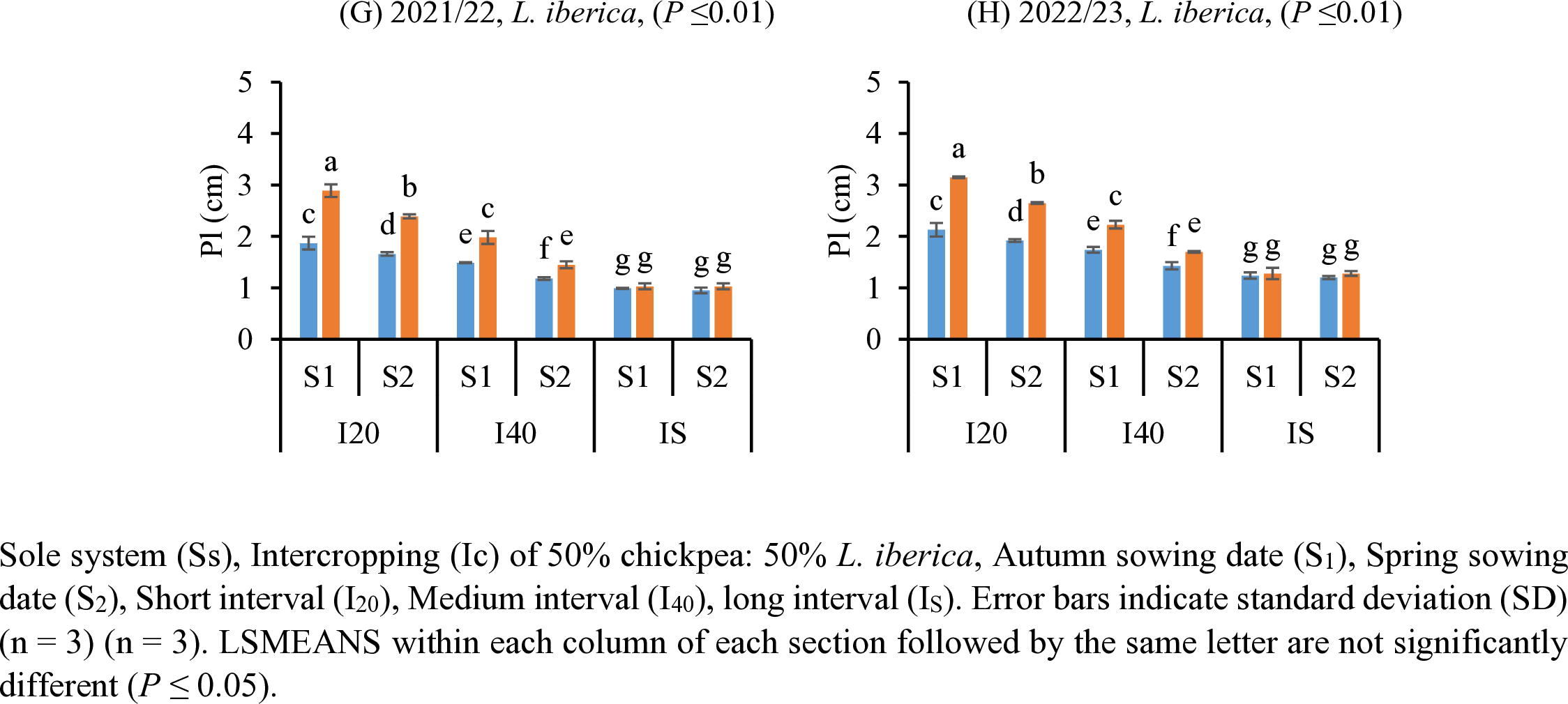

Supplement: S1 File — (ZIP) [file pone.0332264.s001.zip › PACE Corrected/S1 File.tif]

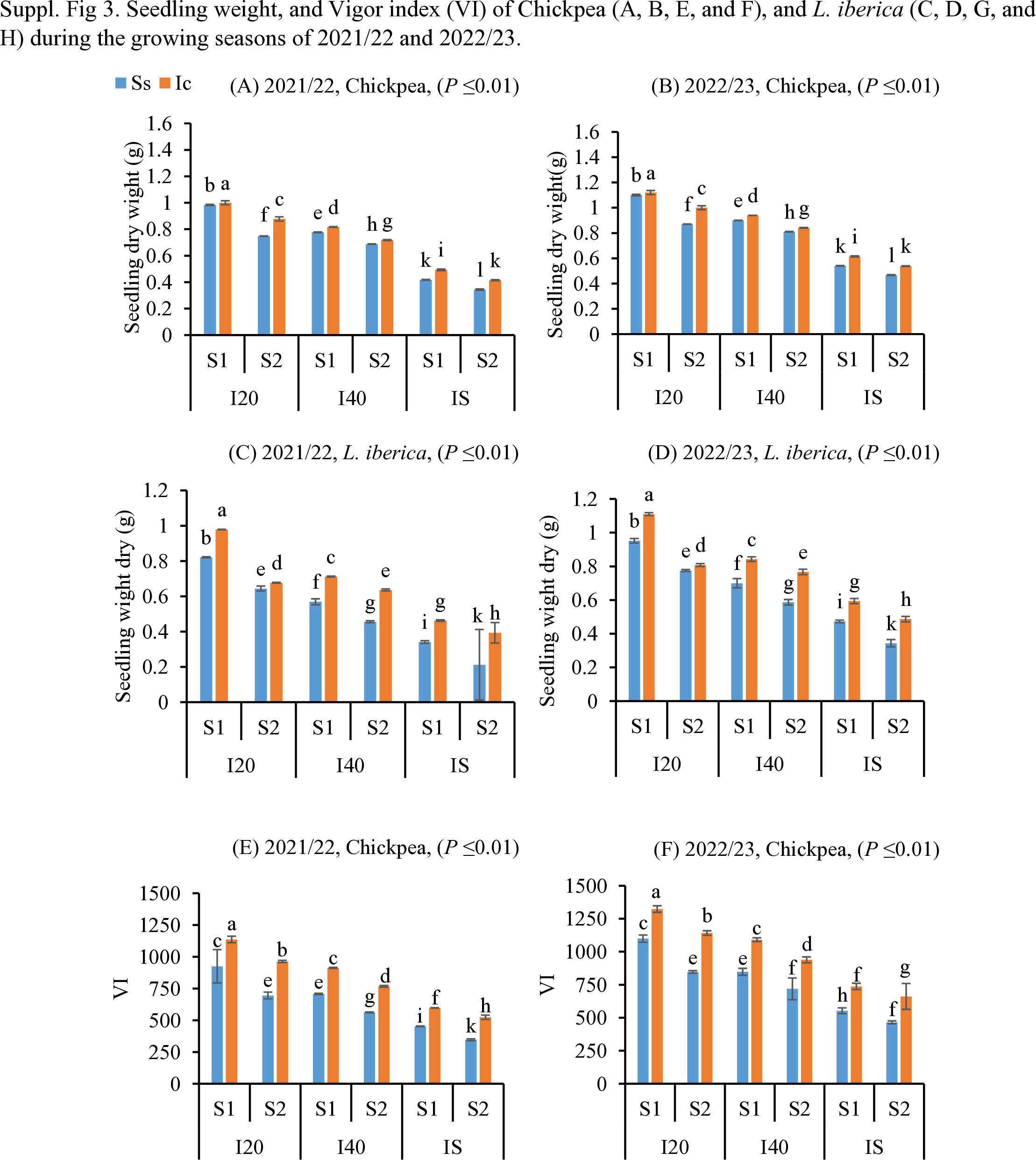

Supplement: S1 File — (ZIP) [file pone.0332264.s001.zip › PACE Corrected/S1 File.tif]

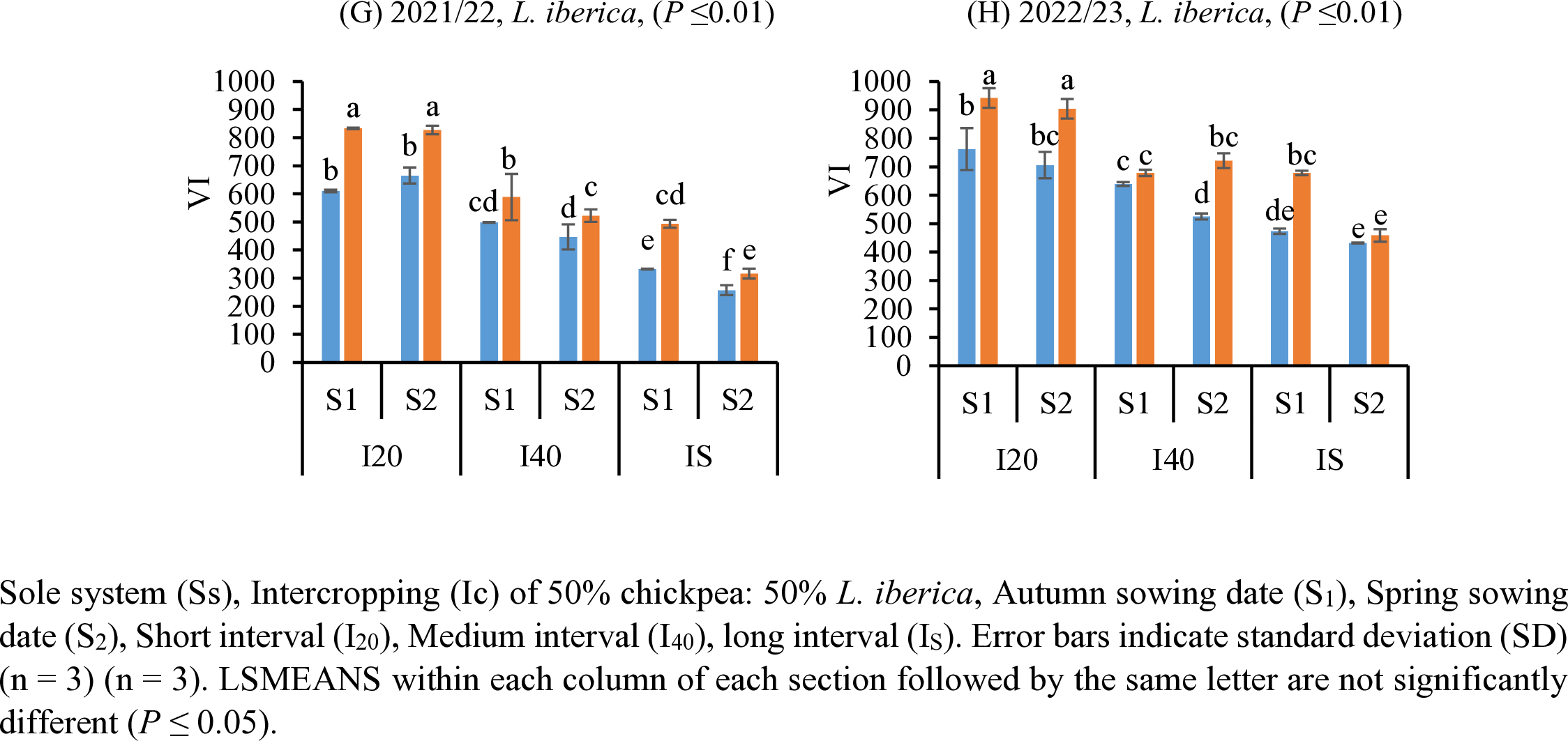

Supplement: S1 File — (ZIP) [file pone.0332264.s001.zip › PACE Corrected/S1 File.tif]

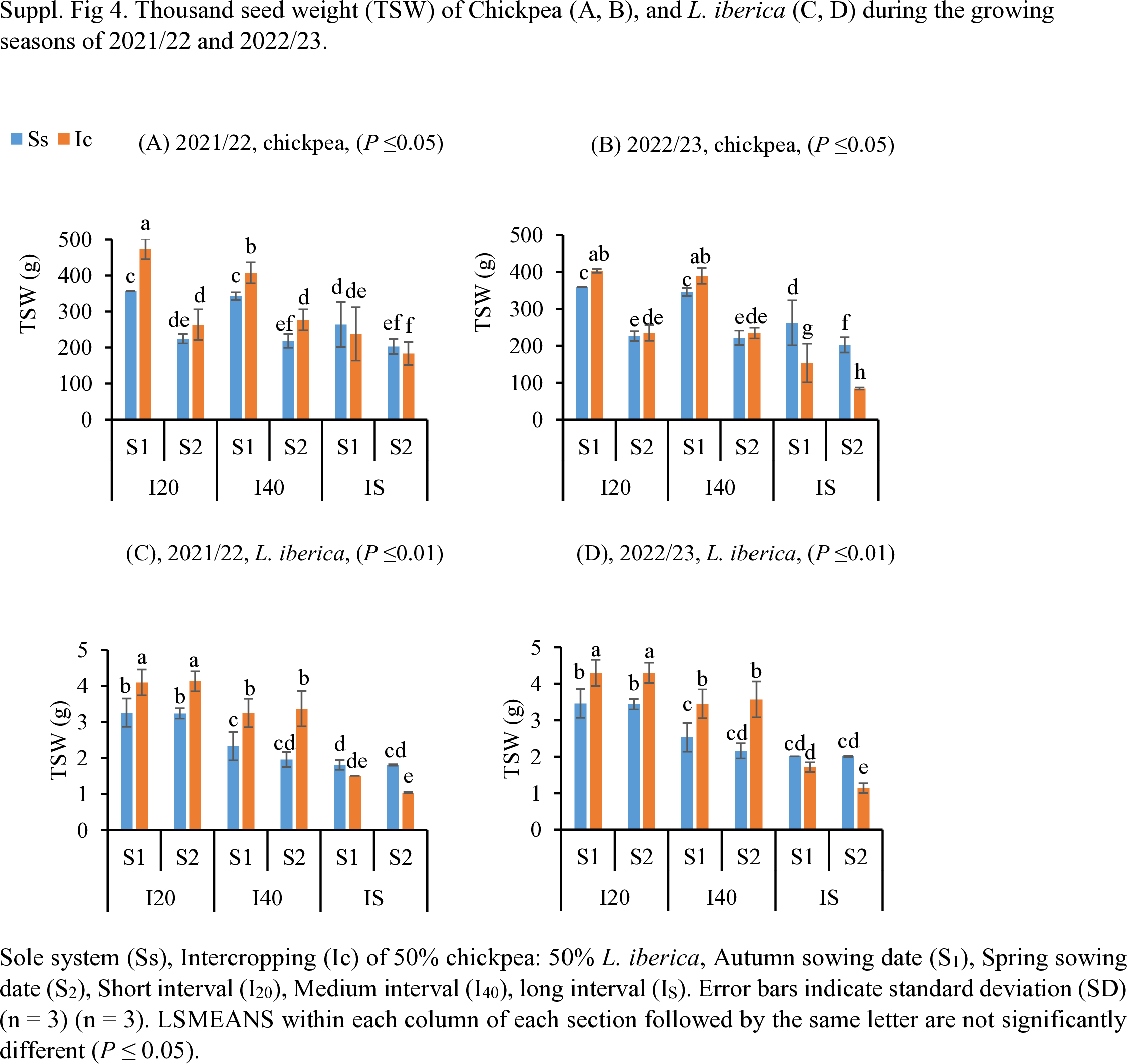

Supplement: S1 File — (ZIP) [file pone.0332264.s001.zip › PACE Corrected/S1 File.tif]

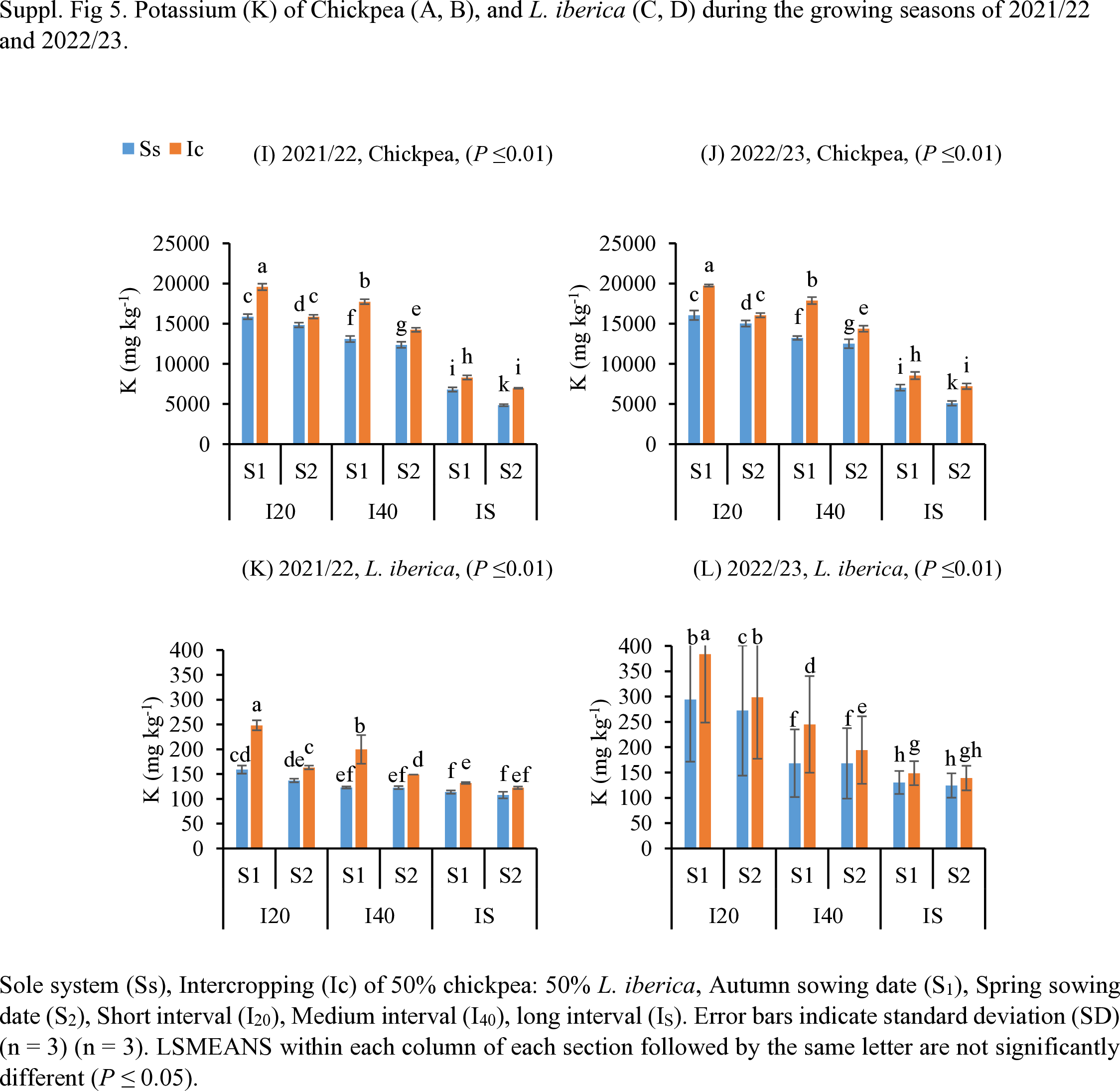

Supplement: S1 File — (ZIP) [file pone.0332264.s001.zip › PACE Corrected/S1 File.tif]

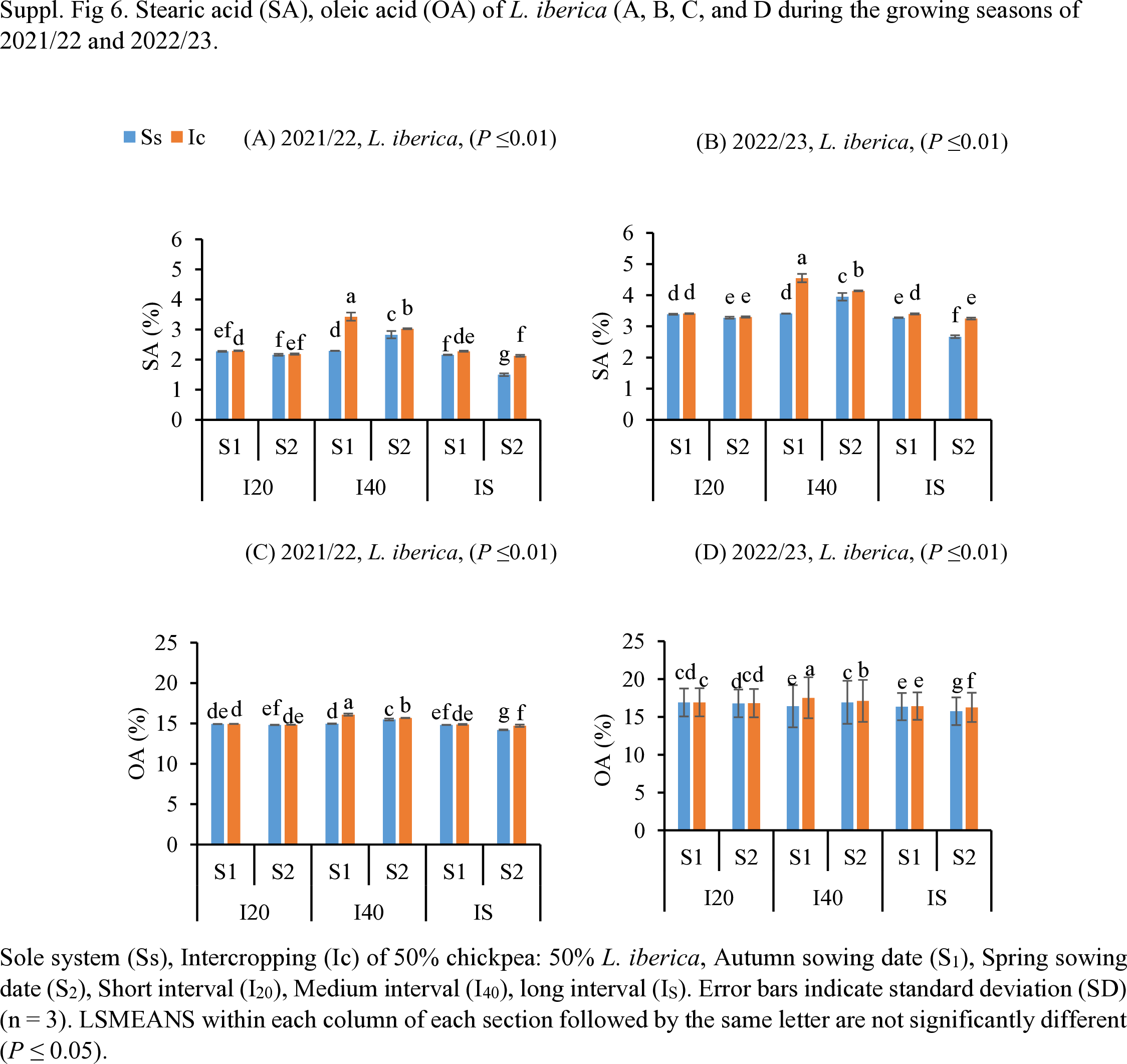

Supplement: S1 File — (ZIP) [file pone.0332264.s001.zip › PACE Corrected/S1 File.tif]
