# Supplementary material for: Seed quality as affected by intercropping of Chickpea and L. iberica
Source: PLoS One. 2025 Oct 30;20(10):e0332264. doi: 10.1371/journal.pone.0332264 (PMC12574852; doi:10.1371/journal.pone.0332264)
Supplement: S1 Table — (DOCX) [file pone.0332264.s003.docx]

Suppl table 1. Monthly rainfall, maximum temperature (Tmax), minimum temperature (Tmin), and relative humidity from November to June in the growing seasons of 2021-22 and 2022-23 in experimental site.

|  |  |  | **Year** |  |  |  |  |  |  |  |
| --- | --- | --- | --- | --- | --- | --- | --- | --- | --- | --- |
|  | **2021-2022** |  |  | |  | **2022-2023** |  |  |  |  |
| **Month** | **Rainfall (mm)** | **Maximum temperature (°C)** | **Minimum temperature (°C)** | **Relative humidity (%)** | **Sunshine (hr)** | **Rainfall (mm)** | **Maximum temperature (°C)** | **Minimum temperature (°C)** | **Relative humidity (%)** | **Sunshine (hr)** |
| November | 13.0 | 18.2 | 4.3 | 53.1 | 162.0 | 4.0 | 28.0 | 6 | 56.5 | 162.3 |
| December | 24.0 | 17.4 | 3.0 | 46.8 | 299.0 | 3.6 | 20.0 | 2 | 49.2 | 298.6 |
| January | 5.0 | 11.4 | 2.4 | 53.6 | 296.8 | 12.8 | 11.0 | -2 | 55.8 | 298.4 |
| February | 3.5 | 11.8 | 5.6 | 41.8 | 317.0 | 35.8 | 12.0 | -2 | 56.3 | 325.9 |
| March | 16.2 | 17.1 | 9.1 | 37.9 | 325.0 | 13.5 | 26.0 | 3 | 51.9 | 304.0 |
| April | 1.3 | 23.6 | 14.3 | 22.1 | 406.4 | 18.6 | 29.0 | 7 | 43.4 | 395.1 |
| May | 2.0 | 28.1 | 19.5 | 26.8 | 427.0 | 1.4 | 34.0 | 9 | 29.5 | 428.3 |
| June | 0.0 | 34.4 | 26.6 | 12.9 | 769.0 | 9.8 | 39.0 | 18 | 26.9 | 435.7 |
| Total | 64.0 |  |  |  |  | 99.5 |  |  |  |  |
| Mean |  | 20.3 | 11.9 | 41.5 | 375.2 |  | 24.9 | 5.1 | 46.9 | 331.0 |
