# Supplementary material for: Seed quality as affected by intercropping of Chickpea and L. iberica
Source: PLoS One. 2025 Oct 30;20(10):e0332264. doi: 10.1371/journal.pone.0332264 (PMC12574852; doi:10.1371/journal.pone.0332264)
Supplement: S3 Table — (DOCX) [file pone.0332264.s005.docx]

Suppl table 3. The combined analysis of variance for the effect of the maternal environment conditions on N, P, and K of *L. iberica* in 2021-22 and 2022-23.

| **Source of**  **variation** | **df** | **N** | **P** | **K** |
| --- | --- | --- | --- | --- |
| Year (Y) | 1 | 14112 ^*^ | 72.0 ^**^ | 77880 ^**^ |
| Irrigation regime (I) | 2 | 19504018 ^**^ | 20125 ^**^ | 84323 ^**^ |
| Y×I | 2 | 0.00 ^ns^ | 0.00 ^ns^ | 23003 ^**^ |
| Block ( Y×I) (Error a) | 12 | 6237 ^*^ | 10.6 ^ns^ | 14867 ^**^ |
| Sowing date (S) | 1 | 20074560 ^**^ | 7980 ^**^ | 15080 ^**^ |
| I×S | 2 | 3133531 ^**^ | 562 ^**^ | 3181 ^**^ |
| Y×S | 1 | 0.00 ^ns^ | 0.00 ^ns^ | 0.00 ^ns^ |
| Y×I×S | 2 | 0.00 ^ns^ | 0.00 ^ns^ | 0.00 ^ns^ |
| Block× (Y×I) | 12 | 3029 ^ns^ | 3.44 ^ns^ | 71.9 ^ns^ |
| Cultivation system (C) | 1 | 4577329 ^**^ | 25312 ^**^ | 31668 ^**^ |
| I×C | 2 | 603096 ^**^ | 1795 ^**^ | 2973 ^**^ |
| Y×C | 1 | 0.00 ^ns^ | 0.00 ^ns^ | 0.00 ^ns^ |
| S×C | 1 | 1351916 ^**^ | 3280 ^**^ | 6844 ^**^ |
| Y×I×C | 2 | 0.00 ^ns^ | 0.00 ^ns^ | 0.00 ^ns^ |
| Y×S×C | 1 | 0.00 ^ns^ | 0.00 ^ns^ | 0.00 ^ns^ |
| I×S×C | 2 | 310926 ^**^ | 468 ^**^ | 1464 ^**^ |
| Y×I×S×C | 2 | 0.00 ^ns^ | 0.00 ^ns^ | 0.00 ^ns^ |
| Error (b) | 24 | 2307 | 9.16 | 97.3 |
| CV (%) |  | 2.30 | 3.70 | 5.44 |

ns, * and **: non-significant and significant at 5 % and 1 % probability levels, respectively. df: degree of freedom, Nitrogen (N), Phosphorus (P), and Potassium (K).
