# Supplementary material for: Seed quality as affected by intercropping of Chickpea and L. iberica
Source: PLoS One. 2025 Oct 30;20(10):e0332264. doi: 10.1371/journal.pone.0332264 (PMC12574852; doi:10.1371/journal.pone.0332264)
Supplement: S4 Table — (DOCX) [file pone.0332264.s006.docx]

Suppl table 4. The combined analysis of variance for the effect of the maternal environment conditions on PA, SA, OA, LA, LNA, and oil content of *L. iberica* in 2021-22 and 2022-23.

| **Source of**  **variation** | **df** | **PA** | **SA** | **OA** | **LA** | **LNA** | **Oil content** |
| --- | --- | --- | --- | --- | --- | --- | --- |
| Year (Y) | 1 | 35.7 ^**^ | 22.5 ^**^ | 49.6 ^**^ | 29.4 ^**^ | 13.6 ^**^ | 46.7 ^**^ |
| Irrigation regime (I) | 2 | 6.66 ^**^ | 4.92 ^**^ | 4.31 ^**^ | 50.9 ^**^ | 177 ^**^ | 618 ^**^ |
| Y×I | 2 | 0.08 ^**^ | 0.00 ^ns^ | 0.48 ^**^ | 1.74 ^ns^ | 4.09 ^*^ | 10.5 ^**^ |
| Block ( Y×I) (Error a) | 12 | 4.49 ^**^ | 0.00 ^ns^ | 9.71 ^**^ | 5.97 ^**^ | 6.88 ^**^ | 2.57 ^**^ |
| Sowing date (S) | 1 | 0.60 ^**^ | 0.36 ^**^ | 0.38 ^**^ | 129 ^**^ | 302 ^**^ | 129 ^**^ |
| I×S | 2 | 0.04 ^*^ | 0.29 ^**^ | 0.30 ^**^ | 0.11 ^ns^ | 35.4 ^**^ | 0.11 ^ns^ |
| Y×S | 1 | 0.00 ^ns^ | 0.00 ^ns^ | 0.00 ^ns^ | 0.17 ^ns^ | 0.00 ^ns^ | 0.17 ^ns^ |
| Y×I×S | 2 | 0.00 ^ns^ | 0.00 ^ns^ | 0.00 ^ns^ | 0.17 ^ns^ | 0.00 ^ns^ | 0.17 ^ns^ |
| Block× (Y×I) | 12 | 0.01 ^ns^ | 0.00 ^ns^ | 0.00 ^ns^ | 0.88 ^ns^ | 1.40 ^ns^ | 0.88 ^ns^ |
| Cultivation system (C) | 1 | 14.0 ^**^ | 2.17 ^**^ | 1.92 ^**^ | 45.6 ^**^ | 163 ^**^ | 45.6 ^**^ |
| I×C | 2 | 2.24 ^**^ | 0.63 ^**^ | 0.61 ^**^ | 0.38 ^ns^ | 39.9 ^**^ | 0.38 ^ns^ |
| Y×C | 1 | 0.00 ^ns^ | 0.00 ^ns^ | 0.00 ^ns^ | 0.00 ^ns^ | 0.14 ^ns^ | 0.00 ^ns^ |
| S×C | 1 | 1.12 ^**^ | 0.11 ^**^ | 0.11 ^**^ | 7.79 ^**^ | 77.6 ^**^ | 7.79 ^**^ |
| Y×I×C | 2 | 0.00 ^ns^ | 0.00 ^ns^ | 0.00 ^ns^ | 0.00 ^ns^ | 0.14 ^ns^ | 0.00 ^ns^ |
| Y×S×C | 1 | 0.00 ^ns^ | 0.00 ^ns^ | 0.00 ^ns^ | 0.62 ^ns^ | 0.14 ^ns^ | 0.62 ^ns^ |
| I×S×C | 2 | 0.10 ^**^ | 0.76 ^**^ | 0.73 ^**^ | 1.88 ^*^ | 45.5 ^**^ | 1.88 ^*^ |
| Y×I×S×C | 2 | 0.00 ^ns^ | 0.00 ^ns^ | 0.00 ^ns^ | 0.62 ^ns^ | 0.14 ^ns^ | 0.62 ^ns^ |
| Error (b) | 24 | 0.01 | 0.00 | 0.00 | 0.53 | 1.12 | 0.53 |
| CV (%) |  | 1.57 | 1.84 | 0.46 | 6.68 | 1.62 | 3.04 |

ns, * and **: non-significant and significant at 5 % and 1 % probability levels, respectively. df: degree of freedom, palmitic acid (PA), stearic acid (SA), oleic acid (OA), linolenic acid (LNA), linoleic acid (LA), Oil content.
