# Supplementary material for: Seed quality as affected by intercropping of Chickpea and L. iberica
Source: PLoS One. 2025 Oct 30;20(10):e0332264. doi: 10.1371/journal.pone.0332264 (PMC12574852; doi:10.1371/journal.pone.0332264)
Supplement: S5 Table — (DOCX) [file pone.0332264.s007.docx]

Suppl table 5. The combined analysis of variance for the effect of the maternal environment conditions on GP, Gr, VI, seedling dry weight, TSW, Pl, Rl, EC, H2O2, MDA, and seed mucilage of chickpea in 2021-22 and 2022-23.

| **Source of**  **variation** | **df** | **GP** | **Gr** | **VI** | **Seedling dry weight** | **TSW** | **Pl** | **Rl** | **EC** | **H2O2** | **MDA** |
| --- | --- | --- | --- | --- | --- | --- | --- | --- | --- | --- | --- |
| Year (Y) | 1 | 98.0 ^**^ | 0.00 ^**^ | 422179 ^**^ | 0.26 ^**^ | 13927 ^**^ | 6.53 ^**^ | 12.2 ^**^ | 19.1 ^**^ | 0.72 ^ns^ | 0.72 ^ns^ |
| Irrigation regime (I) | 2 | 4096 ^**^ | 0.00 ^**^ | 1361920 ^**^ | 1.48 ^**^ | 102094 ^**^ | 9.60 ^**^ | 24.1 ^**^ | 1566 ^**^ | 129628 ^**^ | 3134 ^**^ |
| Y×I | 2 | 0.00 ^ns^ | 0.00 ^ns^ | 3900 ^**^ | 0.00 ^ns^ | 1730 ^**^ | 0.05 ^**^ | 0.00 ^ns^ | 10.3 ^*^ | 0.00 ^ns^ | 0.00 ^ns^ |
| Block ( Y×I) (Error a) | 12 | 1.13 ^ns^ | 0.00 ^**^ | 323 ^ns^ | 0.00 ^ns^ | 2578 | 0.00 ^**^ | 0.02 ^**^ | 14.1 ^**^ | 856 ^ns^ | 0.62 ^ns^ |
| Sowing date (S) | 1 | 102 ^**^ | 0.00 ^**^ | 383559 ^**^ | 0.24 ^**^ | 252616 ^**^ | 3.01 ^**^ | 20.4 ^**^ | 1162 ^**^ | 85605 ^**^ | 2912 ^**^ |
| I×S | 2 | 2.05 ^*^ | 0.00 ^**^ | 23004 ^**^ | 0.01 ^**^ | 15925 ^**^ | 0.20 ^**^ | 0.14 ^**^ | 151 ^**^ | 2483 ^*^ | 417 ^**^ |
| Y×S | 1 | 0.00 ^ns^ | 0.00 ^ns^ | 6.66 ^ns^ | 0.00 ^ns^ | 2.27 ^ns^ | 0.00 ^ns^ | 0.00 ^ns^ | 0.07 ^ns^ | 24.5 ^ns^ | 0.00 ^ns^ |
| Y×I×S | 2 | 0.00 ^ns^ | 0.00 ^ns^ | 230 ^ns^ | 0.00 ^ns^ | 476 ^ns^ | 0.16 ^**^ | 0.00 ^ns^ | 3.07 ^ns^ | 24.5 ^ns^ | 0.00 ^ns^ |
| Block× (Y×I) | 12 | 0.47 ^ns^ | 0.00 ^ns^ | 224 ^ns^ | 0.00 ^ns^ | 1047 | 0.00 ^ns^ | 0.00 ^ns^ | 6.59 ^*^ | 891 ^ns^ | 0.89 ^ns^ |
| Cultivation system (C) | 1 | 1266 ^**^ | 0.00 ^**^ | 829278 ^**^ | 0.06 ^**^ | 1635 ^*^ | 4.89 ^**^ | 29.00 ^**^ | 1560 ^**^ | 79946 ^**^ | 1763 ^**^ |
| I×C | 2 | 108 ^**^ | 0.00 ^**^ | 8277 ^**^ | 0.00 ^**^ | 27223 ^**^ | 0.08 ^**^ | 0.83 ^**^ | 45.2 ^**^ | 9339 ^**^ | 707 ^**^ |
| Y×C | 1 | 0.00 ^ns^ | 0.00 ^ns^ | 2802 ^**^ | 0.00 ^ns^ | 15322 ^**^ | 0.25 ^**^ | 0.00 ^ns^ | 1.89 ^ns^ | 19.5 ^ns^ | 8.95 ^ns^ |
| S×C | 1 | 0.05 ^ns^ | 0.00 ^**^ | 2724 ^**^ | 0.00 ^**^ | 2909 ^**^ | 0.00 ^ns^ | 2.20 ^**^ | 22.3 ^**^ | 10396 ^**^ | 10.6 ^ns^ |
| Y×I×C | 2 | 0.00 ^ns^ | 0.00 ^ns^ | 33.6 ^ns^ | 0.00 ^ns^ | 1295 ^*^ | 0.06 ^**^ | 0.00 ^ns^ | 2.48 ^ns^ | 27.3 ^ns^ | 5.68 ^ns^ |
| Y×S×C | 1 | 0.00 ^ns^ | 0.00 ^ns^ | 94.7 ^ns^ | 0.00 ^ns^ | 0.18 ^ns^ | 0.00 ^ns^ | 0.00 ^ns^ | 0.38 ^ns^ | 0.17 ^ns^ | 4.97 ^ns^ |
| I×S×C | 2 | 4.05 ^**^ | 0.00 ^**^ | 2149 ^**^ | 0.00 ^**^ | 1175 ^*^ | 0.20 ^**^ | 0.34 ^**^ | 32.6 ^**^ | 18225 ^**^ | 32.5 ^*^ |
| Y×I×S×C | 2 | 0.00 ^ns^ | 0.00 ^ns^ | 145 ^ns^ | 0.00 ^ns^ | 494 ^ns^ | 0.16 ^**^ | 0.00 ^ns^ | 0.89 ^ns^ | 0.17 ^ns^ | 7.65 ^ns^ |
| Error (b) | 24 | 0.52 | 0.00 | 291 | 0.00 | 241 | 0.00 | 0.00 | 2.21 | 545 | 9.20 |
| CV (%) |  | 0.85 | 1.79 | 2.15 | 0.86 | 5.67 | 1.52 | 0.74 | 5.17 | 10.2 | 11.9 |

ns, * and **: non-significant and significant at 5 % and 1 % probability levels, respectively. df, degree of freedom, Germination percentage (GP), Germination rate (Gr), Vigor index (VI), Thousand seed weight (TSW); Plumule length (Pl), Radicle length (Rl), Electrical conductivity (EC), Hydrogen peroxide content (H_2_O_2_), and Malondialdehyde (MDA).
