# Supplementary material for: Seed quality as affected by intercropping of Chickpea and L. iberica
Source: PLoS One. 2025 Oct 30;20(10):e0332264. doi: 10.1371/journal.pone.0332264 (PMC12574852; doi:10.1371/journal.pone.0332264)
Supplement: S6 Table — (DOCX) [file pone.0332264.s008.docx]

Suppl table 6. The combined analysis of variance for the effect of the maternal environment conditions on N, P, and K of chickpea in 2021-22 and 2022-23.

| **Source of**  **variation** | **df** | **N** | **P** | **K** |
| --- | --- | --- | --- | --- |
| Year (Y) | 1 | 16897422 ^**^ | 996872 ^**^ | 597689 ^*^ |
| Irrigation regime (I) | 2 | 771077222 ^**^ | 4629844 ^**^ | 631324550 ^**^ |
| Y×I | 2 | 2222 ^ns^ | 14716 ^ns^ | 15022 ^ns^ |
| Block ( Y×I) (Error a) | 12 | 687422 ^ns^ | 121229 ^**^ | 191856 ^*^ |
| Sowing date (S) | 1 | 80222222 ^**^ | 3709180 ^**^ | 74827222 ^**^ |
| I×S | 2 | 3487222 ^**^ | 247870 ^**^ | 810139 ^**^ |
| Y×S | 1 | 0.00 ^ns^ | 0.000 ^ns^ | 0.00 ^ns^ |
| Y×I×S | 2 | 0.00 ^ns^ | 0.000 ^ns^ | 0.00 ^ns^ |
| Block× (Y×I) | 12 | 541111 ^ns^ | 2881 ^ns^ | 126250 ^ns^ |
| Cultivation system (C) | 1 | 33800000 ^**^ | 9796213 ^**^ | 110508889 ^**^ |
| I×C | 2 | 4281667 ^**^ | 265864 ^**^ | 3209306 ^**^ |
| Y×C | 1 | 0.00 ^ns^ | 0.000 ^ns^ | 0.00 ^ns^ |
| S×C | 1 | 9388889 ^**^ | 1608620 ^**^ | 11680556 ^**^ |
| Y×I×C | 2 | 0.00 ^ns^ | 0.000 ^ns^ | 0.00 ^ns^ |
| Y×S×C | 1 | 0.00 ^ns^ | 0.000 ^ns^ | 0.00 ^ns^ |
| I×S×C | 2 | 2537222 ^**^ | 168277 ^**^ | 5588472 ^**^ |
| Y×I×S×C | 2 | 0.00 ^ns^ | 0.00 ^ns^ | 0.00 ^ns^ |
| Error (b) | 24 | 360556 | 5237 | 84583 |
| CV (%) |  | 2.01 | 3.16 | 2.30 |

ns, * and **: non-significant and significant at 5 % and 1 % probability levels, respectively. df: degree of freedom, Nitrogen (N), Phosphorus (P), and Potassium (K).
